# Inducing secondary metabolite production of *Aspergillus sydowii* through microbial co-culture with *Bacillus subtilis*

**DOI:** 10.1186/s12934-021-01527-0

**Published:** 2021-02-12

**Authors:** Yu Sun, Wen-Cai Liu, Xuan Shi, Hai-Zhou Zheng, Zhi-Hui Zheng, Xin-Hua Lu, Yan Xing, Kai Ji, Mei Liu, Yue-Sheng Dong

**Affiliations:** 1 School of Bioengineering, Dalian University of Technology, DalianLiaoning, 116024 China; 2 New Drug Research and Development Center, North China Pharmaceutical Group Corporation and National Microbial Medicine Engineering and Research Center, Shijiazhuang, 050015 Hebei China; 3 CAS Key Laboratory of Microbial Physiological and Metabolic Engineering, Institute of Microbiology, Chinese Academy of Sciences, Beijing, 100101 China; 4 University of Chinese Academy of Sciences, Beijing, 101408 China; 5 Shandong New Time Pharmaceutical Co., Ltd, Shandong, 255000 China

**Keywords:** *Aspergillus sydowii*, *Bacillus subtilis*, Co-culture, Natural products

## Abstract

**Background:**

The co-culture strategy which mimics natural ecology by constructing an artificial microbial community is a useful tool to activate the biosynthetic gene clusters to generate new metabolites. However, the conventional method to study the co-culture is to isolate and purify compounds separated by HPLC, which is inefficient and time-consuming. Furthermore, the overall changes in the metabolite profile cannot be well characterized.

**Results:**

A new approach which integrates computational programs, MS-DIAL, MS-FINDER and web-based tools including GNPS and MetaboAnalyst, was developed to analyze and identify the metabolites of the co-culture of *Aspergillus sydowii* and *Bacillus subtilis*. A total of 25 newly biosynthesized metabolites were detected only in co-culture. The structures of the newly synthesized metabolites were elucidated, four of which were identified as novel compounds by the new approach. The accuracy of the new approach was confirmed by purification and NMR data analysis of 7 newly biosynthesized metabolites. The bioassay of newly synthesized metabolites showed that four of the compounds exhibited different degrees of PTP1b inhibitory activity, and compound **N2** had the strongest inhibition activity with an IC_50_ value of 7.967 μM.

**Conclusions:**

Co-culture led to global changes of the metabolite profile and is an effective way to induce the biosynthesis of novel natural products. The new approach in this study is one of the effective and relatively accurate methods to characterize the changes of metabolite profiles and to identify novel compounds in co-culture systems.

## Background

Natural products (NPs) are an important historical source of many useful drugs and other chemical agents, of which microbial secondary metabolites represent a significant part [1]. Numerous novel secondary metabolites have been isolated from marine fungi and 70–80% of them have good biological activities such as anti-cancer, anti-bacteria, anti-parasite and free-radical scavenging. Moreover, some compounds have been marketed as commercial drugs through clinical research [2]. However, with the progress of scientific research, researchers have found that repeated discoveries of known metabolites are increasing. The fact that the biosynthetic potential has eluded is mostly explained by the observation that many genes are transcriptionally silent under standard culture conditions, causing their products inaccessible [3]. Moreover, analyses of microbial whole genome sequences indicate that microbes contain many thousands of biosynthetic gene clusters, which encode a plethora of compounds that are not identified when cultured under standard laboratory conditions [4]. To overcome these impasses, several approaches have been developed, such as nontargeted metabolic engineering, epigenetic modification, and chemical synthesis. Among such approaches, the co-culture method draws increasing attention to stimulate the production of novel natural products.

Co-culture of different microorganisms can imitate the natural microbial environment, and the silent biosynthetic gene clusters are transcriptionally activated by environmental stimuli [5]. The chemical cues released by other microbes can cause various defense responses, including the changes of mycelial morphology, synthesis of diverse secondary metabolites, and production of extracellular enzymes. Indeed, these activated defensive metabolites can act as chemical cues that can trigger a series of transcriptional activation [6]. Recently, there have been many successful studies on the use of microbial co-culture to induce new secondary metabolites. For instance, Zuck et al. demonstrated that co-culture of *Aspergillus fumigatus* and *Streptomyces peucetius* induced the production of four formyl xanthine analogs that were not generated in pure culture, of which two were compounds with novel structures, and compound **2** showed significant inhibitory activity against several cell lines [7]. Moreover, Wu et al. co-cultured *Bacillus amyloliquefaciens* and *Trichoderma asperellum* and found that the production of antibacterial substances was significantly higher than that in pure culture. When the inoculation ratio was 1:1, the production of specific amino acids was improved [8]. Therefore, co-culture is regarded as a useful research method for effectively inducing the production of metabolites.

*Aspergillus sydowii* can produce various secondary metabolites which are increasingly utilized in pharmaceuticals, food and chemicals, such as endoglucanases with industrial application value, enzymes with inhibitory activity against protein tyrosine phosphatase A of *M. tuberculosis*, sesquiterpenoids with antimicrobial and antiviral activities, and alkaloids with activity against *S. aureus* and *S. epidermidis* [9]. However, the analysis of the whole genome of *A. sydowii* revealed a number of genes for the biosynthesis of compounds that have not been observed when cultured under standard conditions [10]. The previous study has showed that the addition of 5-azacytidine, an epigenetic modifier, to the broth of *A. sydowii* induced the production of the (*S*)-(+)-sydonol which potentiated the insulin-stimulated glucose consumption, suggesting that the metabolites of *A. sydowii* obtained through nontargeted metabolic engineering might be developed into antidiabetic agents [11]. Recently, the enzymes from the protein tyrosine phosphatases (PTPs) superfamily are emerging as potential new drug targets for type 2 diabetes. For example, protein tyrosine phosphatase 1b (PTP1b) [12] is a negative regulator of insulin action in the insulin receptor signaling pathway, SH2-containing protein tyrosine phos-phatase-1 (SHP1) is a negative regulator in signaling pathways, which regulates glucose homeostasis through the modulation of insulin signaling in liver and muscle [13], and Leukocyte common antigen (CD45) is the receptor for some ligands, which can regulate the recruitment of SHP-1 [14]. Some microbial metabolites, such as varic acid analogues from fungi, showed selective inhibitory activities against PTPs [15]. Thus, the activities of metabolites of co-culture on the PTPs and their potential to be used in diabetes treatment is worth expecting.

The conventional method for the study of metabolites in co-culture systems is to separate and purify the compounds corresponding to each peak newly detected in HPLC, and to analyze their structures by means of MS, UV, IR, and NMR [16]. However, the conventional method is inefficient and time-consuming, and only products with high contents can be identified. The elucidation of trace newly biosynthesized metabolites in co-culture systems is still challenging. Furthermore, the overall changes of metabolite profiles during the co-culture cannot be displayed. In these years, metabolomics, which is mainly aided by the advances in analytical technologies, such as high-resolution mass spectrometry (MS), is primarily associated with comprehensive analysis of small-molecule compounds that can be found in biological samples. Some useful tools, for instance, computation-based MS-DIAL [17] and MS-FINDER [18] programs, and the web-based global natural product social molecular network (GNPS) [19] have also been developed to predict the structure of the metabolites. However, in most cases, only one single tool was used in structure predictions, and the accuracy of the predictions is still a concern. In addition, a web-based tool, MetaboAnalyst, which combined multivariate statistics to identify spectral features that are statistically different between two (or more) different sample populations, is useful in the statistical and functional analysis of metabolomic data [20]. It has been reported that these tools have been used in the analysis of the metabolite profile of microorganisms regulated by epigenetics [21]. However, to our best knowledge, there has been no report on the application of MetaboAnalyst in the co-culture of microbes.

In the present study, the fungus *A. sydowii* was co-cultured with the bacterium *B. subtilis*, and an integrated metabolomics approach, composed of MetaboAnalyst, MS-DIAL, MS-FINDER, and GNPS was developed to analyze the MS/MS data of the co-culture. The changes in the metabolite profile were characterized, and the newly biosynthesized compounds were identified. The purification and NMR analysis of part of the newly biosynthesized compounds were performed to verify the accuracy of the new approach. The activities of newly biosynthesized compounds against protein tyrosine phosphatases (PTPs) were also evaluated.

## Results

### Microbial interaction induced changes of the metabolite profile

**Fig. 1:**
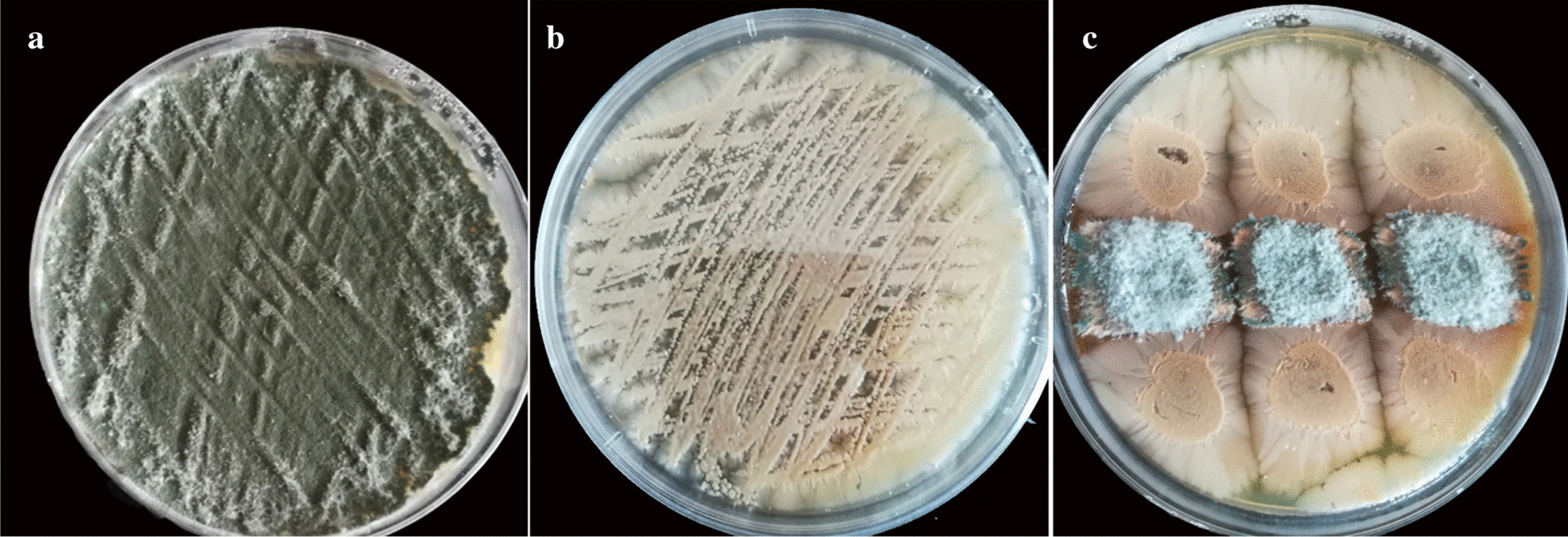
Microorganisms on bran agar plate. **a** Pure culture of *A. sydowii* on Day 12. **b** Pure culture of *B. subtilis* on Day 12. **c** Co-culture of *A. sydowii* and *B. subtilis* on Day 12. *A. sydowii* was in the center and *B. subtilis* was at both sides. A reddish-brown exudate was observed between the two microorganisms

The co-culture of twenty microorganisms with *A. sydowii* on bran medium showed different degrees of induction between the cultures, among which *B. subtilis* could significantly induce *A. sydowii* to produce metabolites (Additional file 1: Fig. S1). After 12 days, the color of the hyphae of *A. sydowii* turned from dark green to light green in co-culture, and red-brown exudate was generated at the junction between *B. subtilis* and *A. sydowii* (Fig. 1). Moreover, a significant deadlock model was observed. This phenomenon indicated that during co-culture, *A. sydowii* and *B. subtilis* generated compounds due to the stress response at the confrontation zone, inhibiting the growth of the other. In order to further explore this phenomenon, we collected the confrontation zone and analyzed the metabolites.

**Fig. 2:**
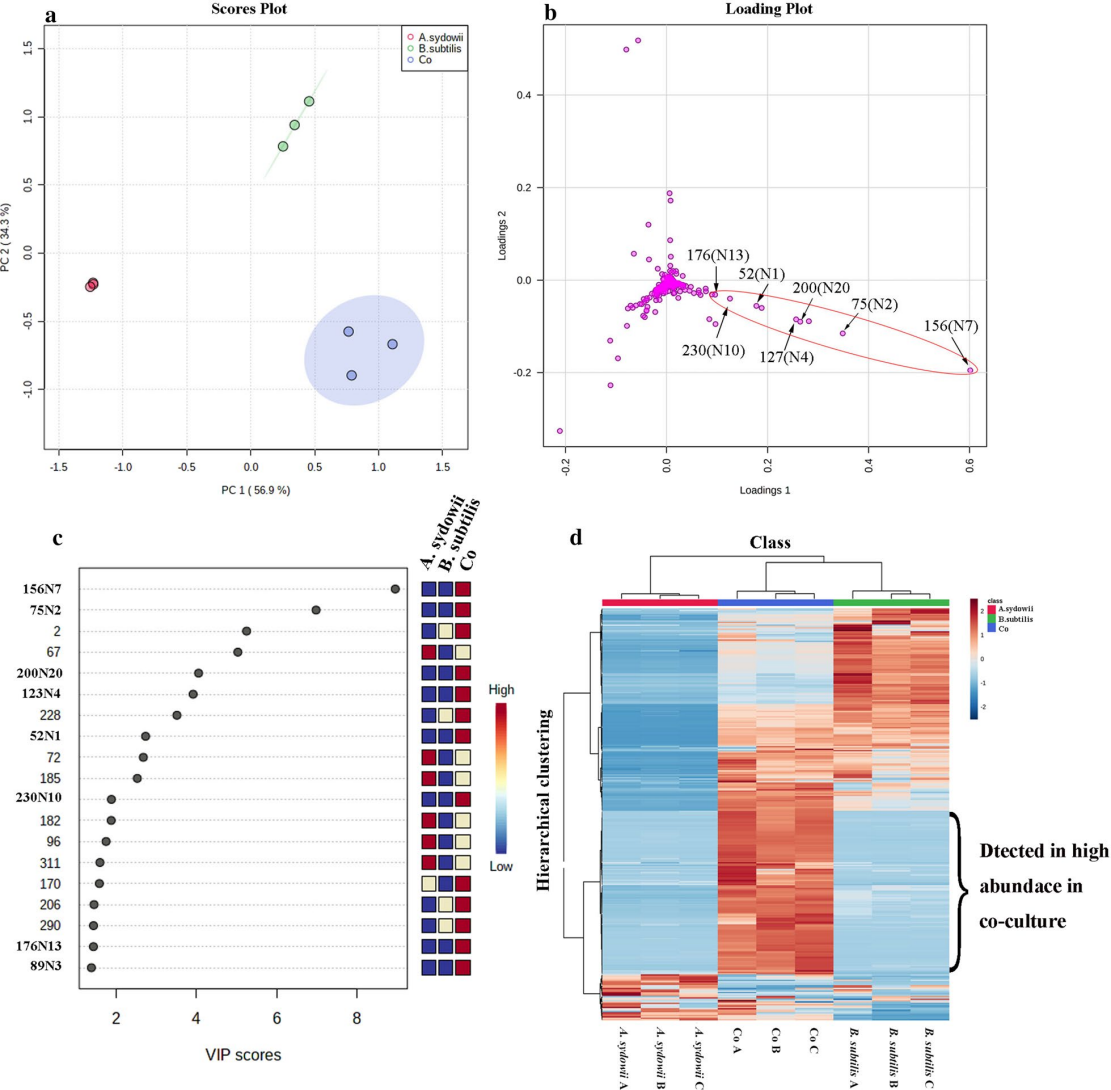
The PLS-DA, VIP and Heatmap of metabolomics data of co-culture and their pure-cultures on Day 12 is shown. **a** The score plot of the data analyzed by LC-HRMS. **b** The loading plot of the data analyzed by LC-HRMS. **c** The top compounds ranked based on the VIP score. The colored boxes on the right indicate the relative concentrations of the corresponding metabolite in each group. **d** Hierarchical clustering analysis (HCA) of the most significantly variable 206 features among the samples corresponding to the three different groups and represented on a heatmap (ranging from red color for high abundance to blue for low abundance). Data was acquired from three independent biological replicates

**Fig. 3:**
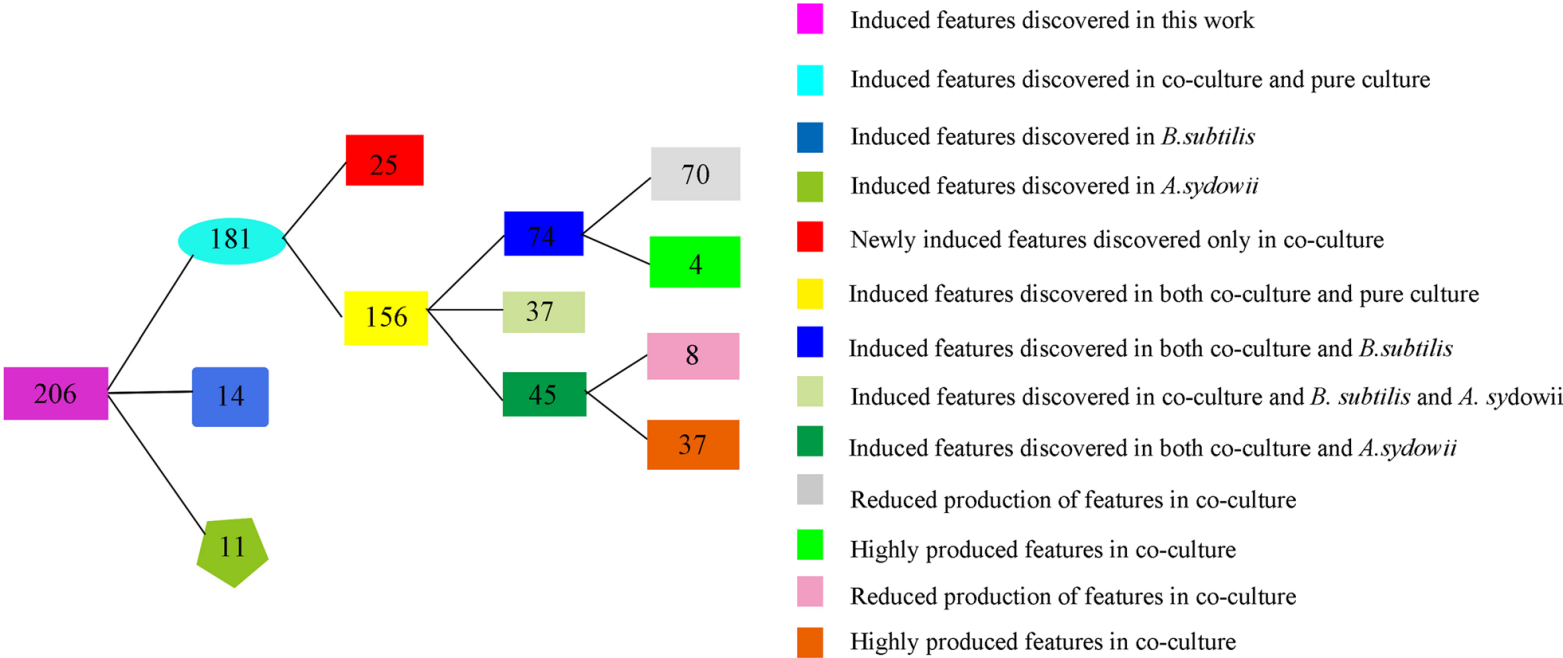
Overview of the number of the induced features in the co-culture of *A. sydowii* and *B. subtilis*

The extracts from the bran medium of co-culture and pure culture were compared by LC–MS/MS, and 206 strong signal features whose intensity was over 10% of the highest intensity peaks were detected. The partial least squares discriminant analysis (PLS-DA) of these peaks revealed the intrinsic variation in the data set. In the score plot, the samples from the co-culture were clearly separated from the two pure cultures, indicating the changes of metabolite profile (Fig. 2a). The heatmap generated by hierarchical clustering analysis (HCA) of these 206 features based on the MS data showed that co-culture caused global changes in the metabolomes (Fig. 2d). The heatmap also revealed that 25 features were identified only in co-culture, indicating that about 12.1% of the candidate features were newly biosynthesized during co-culture. In addition, 156 features were recorded in both pure culture and co-culture. Among them, 70 features in the co-culture system were significantly decreased, while 4 features in the co-culture system were up-regulated when compared with the pure culture of *B. subtilis*. On the contrary, there were only 8 features in the co-culture that were significantly decreased, and 37 features in the co-culture that were up-regulated when compared with the pure culture of *A. sydowii* (Fig. 3). In the loading plot of PLS-DA, the 25 newly biosynthesized features were mainly deviated from the center and clustered into the lower right zone of the plot (Fig. 2b). These features showed good linear correlation. Only the features that had a large contribution to the classification generated by co-culture were distributed on this line. The features that contributed more to the classification were closer to the lower right, while the features that contributed less to the classification were clustered on the upper left of the line and were closer to the origin (Additional file 1: Fig. S2). In the meantime, the variable importance in projection (VIP) score data indicated newly biosynthesized features (**N1**–**N4**, **N7**, **N13,** and **N20**) with monoisotopic mass of m/z 168.4234, 266.1459, 282.1436, 282.4537, 353.1765, 402.1640, and 480.3248, respectively, were ranked in top features detected by VIP score (Fig. 2c). These data indicated that the newly biosynthesized features in co-culture made important contribution to group classification.

### Metabolomics study of newly biosynthesized metabolites in the co-culture

To understand the structure of the newly biosynthesized metabolites, the 25 features were identified with the integrated approach. Here, we demonstrate our results using four annotation levels (Level 1–4). Level 1: the structures were annotated on MS-DIAL linked MS/MS databases by the characteristic product ions and neutral losses; Level 2: the metabolite ions were converted into structural information and the structures were annotated by the structure elucidation tool (MS-FINDER); Level 3: the structures were annotated putatively by the correlation with the known structures with the assistance of the network analysis tool (GNPS); Level 4: the structures were identified by separation, purification and NMR spectrum analysis. Next, the identification of feature **N2** was discussed in detail as an example. The adduct ions of feature **N2** were detected as m/z 265.1459 [M−H]^−^ and m/z 325.2437 [M+CH_3_COOH−H]^−^, suggesting that the monoisotopic mass was 266.1518. The PLS-DA analysis indicated that this feature was detected only in the co-culture and contributed greatly to the cluster. Firstly, the structure of **N2** was identified by MS-DIAL (Level 1), which used deconvolution algorithm to obtain the retention time (RT) and m/z data sets of MS/MS data. The compounds were then annotated by comparing the characteristic products and neutral losses of features with the public MS/MS databases [22]. As no structural candidate was obtained from Level 1, the structure of **N2** was annotated through MS-FINDER (Level 2), which was embedded in MS-DIAL software version 3.90. MS-FINDER was a strategy for computational MS/MS fragmentations. In this software, all isomer structures of the predicted formula were retrieved from metabolome databases, and the structure was predicated based on a combined weighting score considering bond dissociation energies, mass accuracies, fragment linkages, and nine rules of hydrogen rearrangement proposed during bond cleavages in low-energy collision-induced-based fragmentation [18]. After comparing in silico spectra and the structures provided by MS-FINDER, **N2** was identified as sydonic acid based on the fact that the mass peaks (m/z 265.1459, 253.4632, 249.1126, 180.0446, 137.0338, and 93.0326) matched well with the MS/MS database (Additional file 1: Fig. S3) with the lowest mass error of 1.0942 ppm and the highest structure score of 7.55. Similarly, among the other 20 features, 5 features were identified through Level 1 process and 15 features were identified through Level 2 process. The detailed information of the metabolites was summarized in the Table 2. There were 5 major classes of metabolites induced by co-culture, including sesquiterpenes, macrolides, esters, polyketides, and flavonoids. These metabolites of microorganism, are usually not generated in the normal condition of microorganisms, and can only be synthesized under certain stress. These compounds were reported to participate in the defense and communication between microbial cells, promote metabolism, and have a certain bacteriostatic effect [23].

**Fig. 4:**
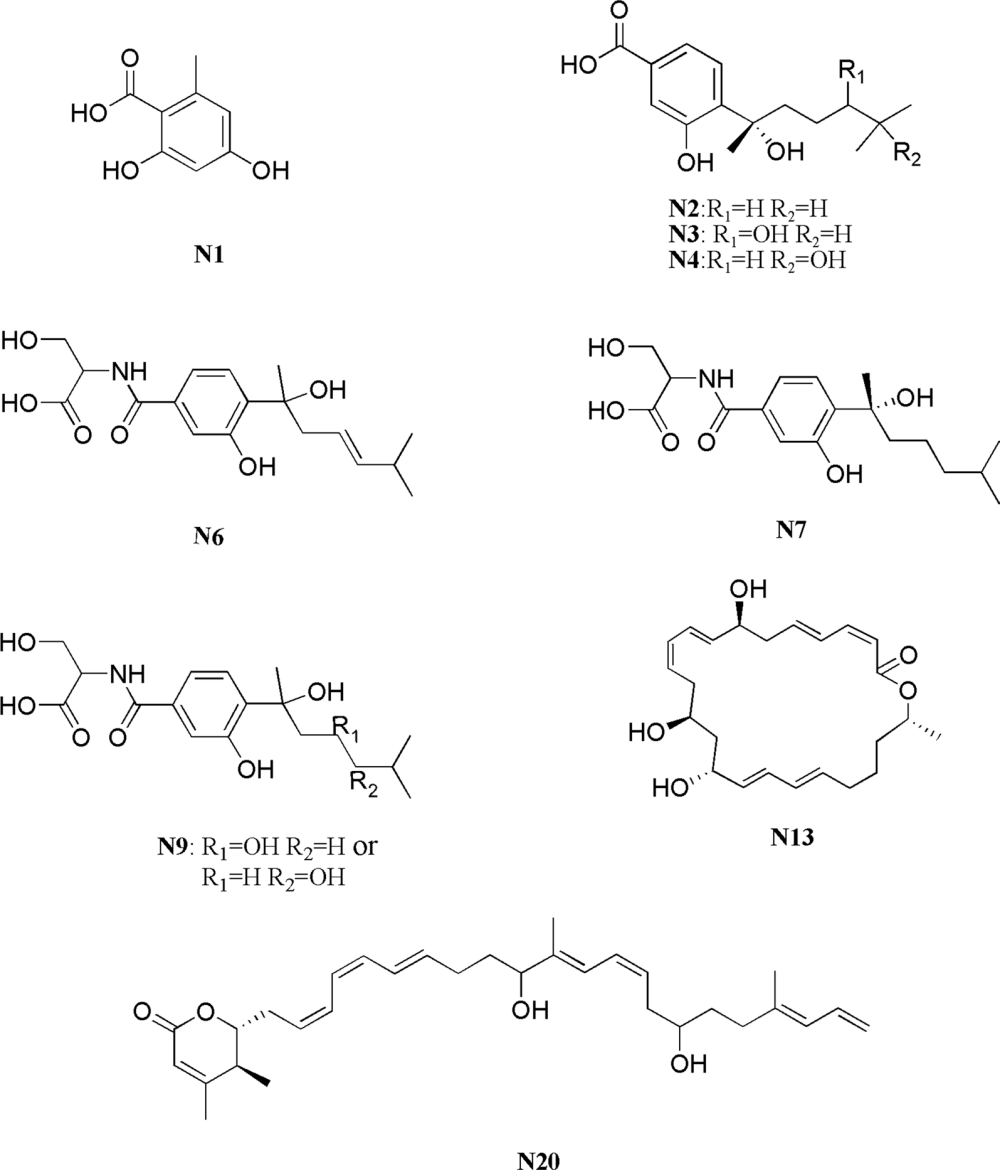
Structures of the compounds

In order to verify the validity of the identification approach, five compounds (**N1**, **N2**, **N3**, **N4,** and **N13**) which had higher VIP scores in PLS-DA analysis indicating larger contributions to the cluster, were isolated and purified through silica gel column chromatography, ODS column chromatography and preparative HPLC from the confrontation zone of the co-culture. According to the results, **N1** (20 mg), **N2** (60 mg), **N3** (19 mg), **N4** (13 mg), and **N13** (21 mg) with purity over 95% were obtained. After analyzing of the NMR data, the compounds were identified as Orsellinic acid (**N1**) [24], Sydonic acid (**N2**) [25], (7*S*)-(−)-10-Hydroxysydonic acid (**N3**) [23], (*R*)-(−)-Hydroxysydonic acid (**N4**) [26], and Macrolactin A (**N13**) [27], which were consistent with those identified by the approach above, suggesting the credibility of the approach. The structures of the 7 compounds were shown in Fig. 4 and the detailed NMR data were provided in Additional file 1: compounds information.

### Identification of the novel metabolites in the co-culture

There were still 4 features (**N6**, **N7**, **N9,** and **N20**) that did not match any features in the public MS/MS spectrum library. To elucidate the structures of these potential novel metabolites generated through co-culture, MS/MS data were analyzed with Level 3 process which was assisted by GNPS platform and manual dereplication. The GNPS approach can capture similar structures and analog features into the same cluster regardless of retention time in the LC–MS.

**Fig. 5:**
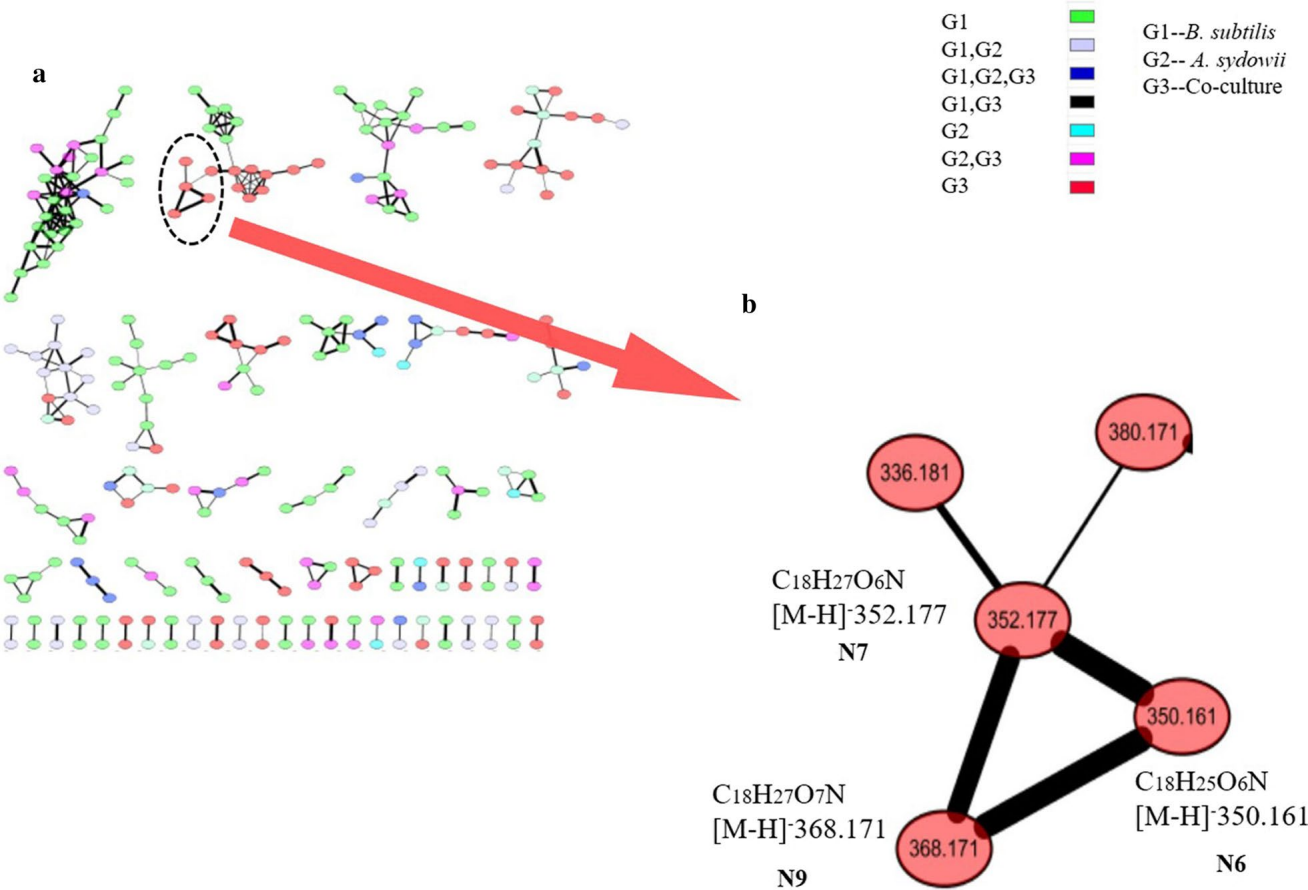
Molecular network analysis of newly discovered features in the co-culture. **a** A constructed subnetwork to dereplicate co-culture and pure culture; **b** two newly identified features (m/z 369.171, 351.161) and the confirmed feature (m/z 353.177) were clustered together

The GNPS data indicated that three induced features, including **N6** (m/z 350.1610), **N7** (m/z 352.1765), and **N9** (m/z 368.1713) were clustered, suggesting that these features have very close structural relationships (Fig. 5). As none of the three features was identified within the LC–MS/MS database, compound **N7**, with the highest content in LC–MS data, was separated and purified. Compound **N7** was obtained as white powder. The UV absorption was at 213 nm, 254 nm, and 298 nm. The molecular formula C_18_H_27_O_6_N was indicated by the ESI-HRMS at m/z 354.1908 [M+H]^+^ (calculated as 354.1872), indicating 6 degrees of unsaturation. The ^1^H NMR and ^13^C NMR of **N7**, **N2** and the reference data of **N2** [25] were shown in Table 1. The ^13^C NMR and HMQC spectra indicated the presence of a total of 18 carbon signals attributable to one carboxyl carbon at δc 172.05 (C-10); one ketone carbon δc 166.14 (C-7); three methyl groups at δc 28.48 (C-8′), δc 22.57 (C-6′) and δc 22.37 (C-7′); four methylene groups at δc 61.35 (C-9), 41.60 (C-2′), 21.33(C-3′) and 38.78(C-4′); five methines including δc 22.37 (C-7′), three aromatic carbons at δc 126.64 (C-6), δc 117.47 (C-5) and δc 115.10 (C-3), one oxygenated carbon at δc 55.53 (C-8); four quaternary carbons including three aromatics at δc 154.74 (C-2), δc 135.86 (C-1) and δc 133.50 (C-4), one oxygenated atom at δc 75.04 (C-1′).

**Table 1.** ^1^H and ^13^C NMR spectral data for compound N7 in DMSO-*d6*, N2 in CD_3_OH and the reference data of sydonic acid in CD_3_OH (500 MHz for ^1^H NMR and 125 MHz for ^13^C NMR)

No.	N7		N2		Sydonic acid	
	δ_C_	δ_H_ (*J* in Hz)	δ_C_	δ_H_ (*J* in Hz)	δ_C_	δ_H_ (*J* in Hz)
1	135.9		137.9		138.0	
2	154.7		157.0		156.9	
3	115.1	7.24 (1H, d, 1.35)	118.7	7.37 (1H, d, 1.6)	118.6	7.36 (1H, d, 1.6)
4	133.5		131.6		131.6	
5	117.5	7.28 (1H, dd, 1.35, 8.05)	121.5	7.44 (1H, dd, 1.6, 7.9)	121.5	7.43 (1H, dd, 1.6, 7.9)
6	126.6	7.35 (1H, d, 8.05)	127.7	7.25 (1H, d, 7.9)	127.7	7.25 (1H, d, 7.9)
7	166.1		169.9		169.9	
8	55.5	4.42 (1H, m)				
9	61.4	3.77 (2H, m)				
10	172.1					
1′	75.0		78.0		77.9	
8′	28.5	1.51 (3H, s)	28.9	1.59 (3H, s)	29.0	1.59 (3H, s)
2′	41.6	1.94 (1H, m)	43.7	1.94 (1H, m)	43.6	1.94 (1H, ddd, 4.5, 12.2, 13.7)
		1.66 (1H, m)		1.77 (1H, m)		1.77 (1H, ddd, 4.8, 11.6, 13.7)
3′	21.3	0.99 (1H, m)	22.9	1.29 (1H, m)	22.9	1.18 (1H, m)
		1.27 (1H, m)		1.34 (1H, m)		1.33 (1H, m)
4′	38.8	1.05 (2H, m)	40.4	1.13 (2H, m)	40.4	1.13 (2H, m)
5′	27.3	1.43 (1H, m)	28.9	1.47 (1H, m)	28.8	1.47 (1H, m)
6′	22.6	0.77 (6H, d, 6.6)	22.8	0.81 (6H, d, 6.4)	22.9	0.81 (6H, d, 6.5)
7′	22.4		23.0		23.0	
N–H		8.15 (1H, d, 7.6)				

**Fig. 6:**
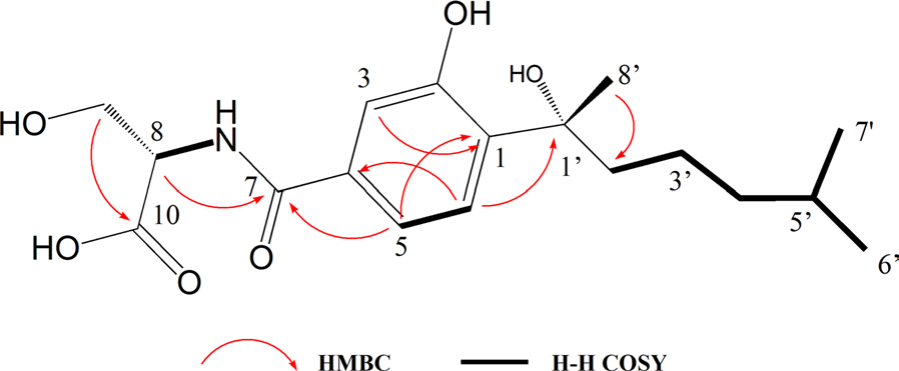
The key COSY and HMBC of compound **N7**

The inspection of the ^1^H NMR spectrum indicated the presence of δ_H_ 7.35 (d, *J* = 8.05 Hz, H-6), 7.28 (dd, *J* = 8.05, 1.35 Hz, H-5) and 7.24 (d, *J* = 1.35 Hz, H-3), which was a typical spectrum of 1,3,4-trisubstituted benzene ring. This sub-structure was confirmed by the relationship between H-5 (δ_H_ 7.28) to H-6 (δ_H_ 7.35) in ^1^H–^1^H COSY, and H-3 (δ_H_ 7.24) to C-1 (δc 135.86), H-5 (δ_H_ 7.28) to C-1, and H-6 (δ_H_ 7.35) to C-4 (δc 133.5) in HMBC. The correlations from H-7′ (δ_H_ 0.77) to H-2′ (δ_H_ 1.94, 1.66) in ^1^H–^1^H COSY indicated the existence of a hexane substructure. The HMBC relationship between H-5 (δ_H_ 7.28) with C-7 (δc 166.14) and H-6 (δ_H_ 7.35) with C-1′ (δc 75.04) implied the linkage of C-4 to the C-1 position. The position of the amine bond was determined by the relationship of H-8 to C-7 in HMBC (Fig. 6; Additional file 1: Figs. S4–S10).

**Fig. 7:**
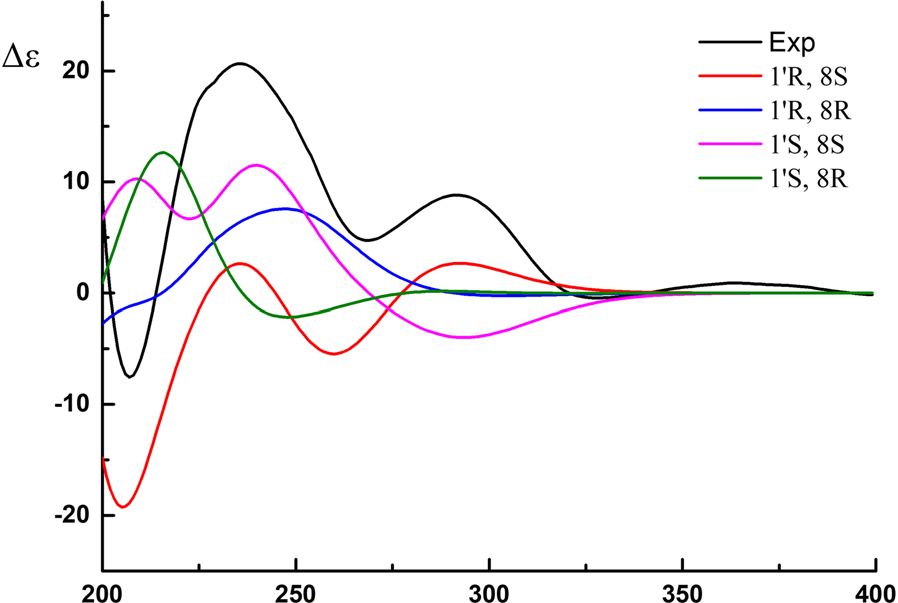
Calculated and experimental ECD spectrums of compound **N7** (black, experimental in MeOH; red, blue, pink, green, calculated at the B3LYP/6-31G (d) //B3LYP/6-31G (d, p) level in MeOH)

The absolute configuration of **N7** was deduced based on the comparison of experimental data and calculated ECD curves by Gaussian 09. The conformers were optimized using DFT at the B3LYP/6-31G (d) level in methanol. The energies were calculated through the TDDFT methodology at the B3LYP/6-31G (d, p) level in MeOH with PCM model (Additional file 1: Figs. S11–S14; Tables S1–S8). The calculated CD spectrum of **N7** (1′R, 8S) agreed well with the experimental CD curve (Fig. 7), indicating that absolute configuration of **N7** was 1′R, 8S, and was named as Serine sydonate.

**Fig. 8:**
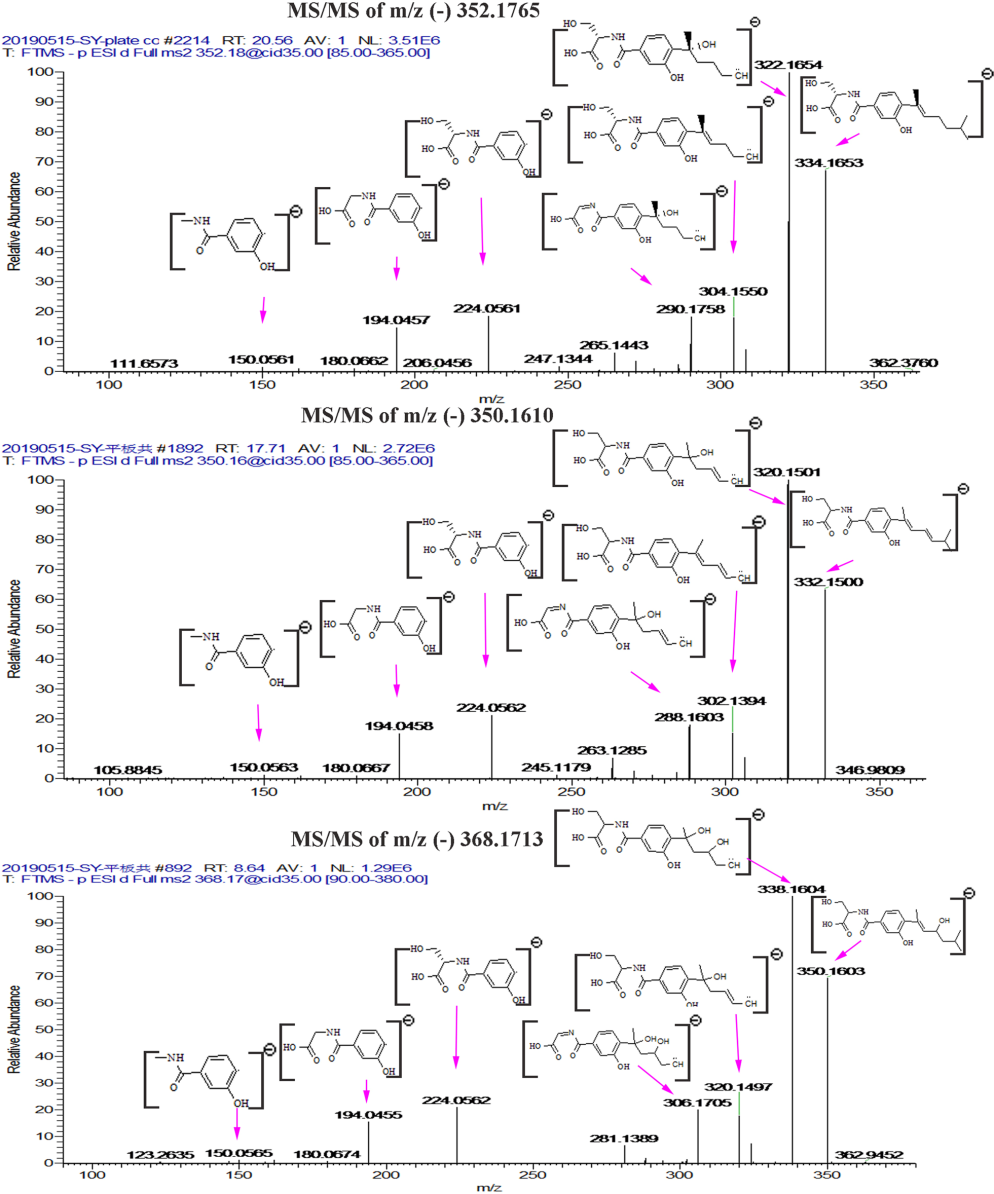
Annotated MS/MS spectrum of m/z 369.1713, 351.1610 and the confirmed feature (m/z 353.1765) acquired by LTQ-Orbitrap-XL in negative mode

The structures of compounds **N6** and **N9** were mainly determined by the LC–MS/MS data from the negative-ion mode and compared with **N7**. Comparison of the fragment ions of the compounds showed some common fragments of m/z 224.0562, m/z 194.0458 and m/z 150.0563, indicating that these features had the same backbone structure and belonged to a series of structural derivatives (Additional file 1: Fig. S15). The structures of compounds **N6** and **N9** were determined by comparing the negative-ion mode LC–MS/MS data with **N7** because these three compounds shared similar MS/MS pattern and the content of **N6** and **N9** was low in broth. For the LC–MS/MS data of **N7**, the abundant fragments were m/z 334.1656 and 322.1660, which arose from molecular ion m/z 352.1765 by the loss of H_2_O (18 Da) and two methyl groups (30 Da), respectively. The fragments of m/z 304.1557 and 290.1758 were generated by further facile loss of H_2_O (18 Da) and CH_3_OH (32 Da) from the fragment of m/z 322.1654, respectively. The fragments of m/z 224.0562, m/z 194.0458 and m/z 150.0563 indicated the substructure of serine substituted hydroxy-benzoic acid. The monoisotopic mass of **N6** was 350.1610 [M−H]^−^, which was common neutral loss of 2 Da of **N7**, indicating that the compound **N6** was most likely the dihydrogen product of **N7**. The fragments of m/z 224.0562, m/z 194.0458 and m/z 150.0563 suggested the existence of the substructure of serine substituted hydroxy-benzoic acid in **N6**. The stable m/z 302.1394 fragment, which represented the conjugated olefin structure formed by the dehydroxylation of hydroxyl methylheptane, suggested that the double bond was located at the 4′ position. Thus, compound **N6** was determined as 4′-alkenyl serine sydonate. For compound **N9**, the fragments of m/z 224.0562, m/z 194.0458 and m/z 150.0563 were also the characteristics of the substructure of serine substituted hydroxy-benzoic acid. The residue mass was 18 Da higher than that of **N6**, suggesting that **N6** was the dehydration product of **N9**. The stable m/z of 302.1394 in **N6** and m/z of 320.1497 in **N9** also indicated that dehydration occurred in the substructure of hydroxyl methylheptan. Thus, the structure of **N9** was determined as hydroxyl serine sydonate. However, whether this hydroxyl group was located at 4′ or 5′ position could not be determined by LC–MS/MS data alone (Fig. 8). **N6** and **N9** (4′-hydroxyl or 5′-hydroxyl serine sydonate) were found to be novel compounds after database searching.

**Fig. 9:**
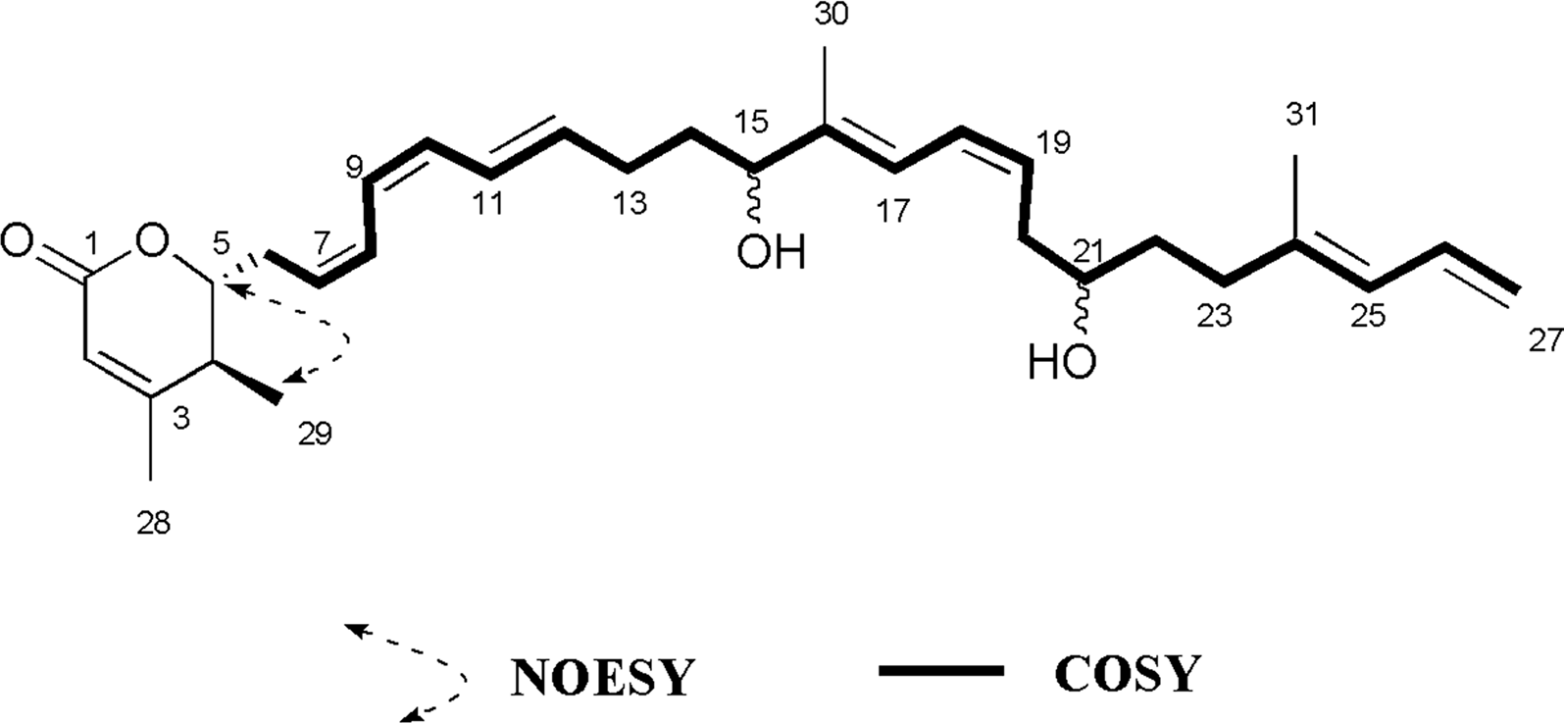
The key COSY and NOESY of compound **N20**

As compound **N20** cannot be connected with the other metabolites in Level 3, it was forwarded to Level 4 for direct isolation and purification. This compound was isolated as pale-yellow creamy solid. The molecular formula C_31_H_44_O_4_ was indicated by the ESI-HRMS at m/z 503.3134 [M+Na]^+^ (calculated for 480.3240), indicating 10 degrees of unsaturation. The UV absorption was at 212 nm and 273 nm. The ^1^H NMR (DMSO-*d6*, 500 MHz) and ^13^C NMR (DMSO-*d6*, 125 MHz) data were provided in the Additional file 1: compounds information. The 1D NMR data were consistent with the data of the known compound Macrolactin U identified by Xue et al. [28], and its relative configuration was 4*S*, 5*S*. However, the methyl group H_3_-29 (δ 1.10, d) and H_2_-6 (δ 2.56, m) were defined as *trans* due to the NOESY relationship between H_3_-29 and H-5 (δ 4.25, m) in compound **N20** (Additional file 1**:** Fig. S16-S23). Thus, the relative configuration of C-4 and C-5 were *S* and *R*, respectively, (Fig. 9), which was different from *S* and *S* in Macrolactin U. Therefore, compound **N20** was identified as the isomer of Macrolactin U and named as Macrolactin U′, which is also a novel compound. Unfortunately, the absolute configuration of **N20** was not determined yet as no obvious difference was identified between ECD and crystal of **N20**, which could not be obtained due to the limited amount of the compound.

Thus, a total of 25 features induced only in the co-culture were identified by the combination of the computational approach (MS-DIAL), the web-based tools (GNPS and MetaboAnalyst) with chemical isolation and purification (Table 2, Additional file 1: Fig. S24). Four of the features were novel metabolites, including two compounds confirmed by NMR. Five known compounds were also purified to verify the validity of the approach.

**Table 2:** List of induced features only in the co-culture of *A. sydowii* with *B. subtilis* analyzed by LC-HRMS

No.	Observed m/z; RT (min)	Calculated m/z	Δ mass (ppm)	Molecular formula	Name	Classification	Identification method
**N1**	*168.4252/12.01*	*168.4253*	*1.1023*	*C*_*8*_*H*_*8*_*O*_*4*_	*Orsellinic acid*	*Sesquiterpene*	*Level 2, 4*
**N2**	*266.1393/30.79*	*266.1418*	*1.0122*	*C*_*15*_*H*_*22*_*O*_*4*_	*Sydonic acid*	*Sesquiterpene*	*Level 2, 4*
**N3**	*282.1412/13.2*	*282.1417*	*1.5212*	*C*_*15*_*H*_*22*_*O*_*5*_	*(7S)-(-)-10-Hydroxysydonic acid*	*Sesquiterpene*	*Level 2, 4*
**N4**	*282.4545/14.01*	*282.4548*	*0.7386*	*C*_*15*_*H*_*22*_*O*_*5*_	*(R)-(−)-Hydroxysydonic acid*	*Sesquiterpene*	*Level 2, 4*
N5	337.3712/25.88	337.3713	0.1517	C_18_H_27_NO_5_	Neostemodiol	Alkaloid	Level 2, 3
N6	351.1683/12.73	351.1682	0.3528	C_18_H_25_NO_6_	Hydroxy serine sydonate	Sesquiterpene	Level 3
**N7**	*353.1858/20.82*	*353.1855*	*1.7889*	*C*_*18*_*H*_*27*_*NO*_*6*_	*Serine sydonate*	*Sesquiterpene*	*Level 4*
N8	367.4195/22.65	367.4193	0.1416	C_19_H_29_NO_6_	Piperidinyl acetic acid ethyl ester	Ester	Level 1
N9	369.1772/31.44	39.1764	3.0703	C_18_H_27_NO_7_	3′-Alkene serine sydonate	Sesquiterpene	Level 3
N10	381.1745/31.44	381.1747	2.1038	C_19_H_27_NO_7_	Bruceolline	Alkaloid	Level 2, 3
N11	395.1978/22.26	395.1974	0.2357	C_20_H_29_NO_7_	Ruwenine	Macrolide	Level 1
N12	398.0976/11.57	398.0978	0.1637	C_21_H_18_O_8_	Auramycinone	Antibiotic	Level 2
**N13**	*402.1678/19.82*	*402.1679*	*0.2387*	*C*_*24*_*H*_*34*_*O*_*5*_	*Macrolactin A*	*Macrolide*	*Level 2, 4*
N14	424.1189/20.42	424.1191	1.7654	C_23_H_20_O_8_	Dehydrovillosin	Polyketide	Level 1
N15	424.1373/11.86	42.1379	1.0165	C_20_H_24_O_10_	Rutarin	Glucoside	Level 2
N16	426.0929/14.44	426.0933	2.3088	C_22_H_18_O_9_	Maggiemycin	Polyketide	Level 2
N17	436.1373/14.3	436.1377	0.9653	C_21_H_24_O_10_	Phlorizin	Antioxidant	Level 1
N18	444.1993/20.07	444.1996	0.8754	C_21_H_32_O_10_	Penstemide	Ester	Level 2
N19	452.1235/14.43	452.1236	0.4373	C_21_H_24_O_11_	Glucuronide	Ester	Level 1
**N20**	*480.3264/39.51*	*480.3265*	*0.8647*	*C*_*31*_*H*_*44*_*O*_*4*_	*Macrolactin U′*	*Macrolide*	*Level 4*
N21	502.2553/28.94	502.2559	1.3436	C_28_H_38_O_8_	Cavipetin	Ester	Level 2
N22	514.2476/18.41	514.2473	2.0741	C_31_H_34_N_2_O_5_	Telmisartan	Aromatic	Level 2
N23	558.2675/15.84	558.2679	1.4823	C_27_H_42_O_12_	Valeriotriate B	Ester	Level 2
N24	601.3283/38.12	601.3285	1.0284	C_34_H_43_N_5_O_5_	Methylurea	Ester	Level 2
N25	706.3683/20.78	706.3689	1.7847	C_42_H_50_N_4_O_6_	Tetrastachynine	Peptide	Level 2

The italicized values refer to compounds which were obtained through separation and purification

### Biological activity assay

The isolated compounds **N1**–**N4**, **N7**, **N13** and **N20** were evaluated for their anti-nematode activity and antidiabetic activity. Among the compounds, compound **N3** showed a certain degree of anti-nematode activity with an IC_50_ of 50 μM. Furthermore, compounds **N2**–**N4**, **N7** and **N13** exhibited potent activity against SHP1 and PTP1b, both of which are targets for the development of diabetes (Table 3). In addition, compounds **N7** and **N13** displayed inhibition activities against CD45 with IC_50_ values of 16.0 μM and 17.9 μM, respectively.

**Table 3:** Activities of compounds

	IC_50_ (μM)		
	PTP1b	SHP1	CD45
**N1**	> 20	> 20	> 20
**N2**	7.97 ± 0.24	8.35 ± 0.35	> 20
**N3**	15.88 ± 0.13	> 20	> 20
**N4**	> 20	15.72 ± 0.11	> 20
**N7**	14.18 ± 0.21	11.68 ± 0.08	16.03 ± 0.38
**N13**	> 20	14.61 ± 0.39	17.89 ± 0.92

## Discussion

Microbial metabolites have always been considered as a very important source of new drugs, due to their various biological activities such as anti-bacteria, anti-oxidation, and anti-tumor. When two microorganisms are co-cultured, new metabolites can be biosynthesized by one or both microorganisms as a result of interspecific crosstalk or induction by biochemical signaling molecules [29]. For example, Akone et al. [30] co-cultured *Chaetomium* sp. with *B. subtilis*, obtaining 5 new compounds and 7 known compounds. However, how to characterize the overall changes in the metabolite profile induced by co-culture and to identify the newly biosynthesized metabolites is still a complicated and challenging task.

The fragmentation pattern in the MS/MS spectrum represents a specific feature of a certain compound. The structures and chemical properties of the molecules determine the observable fragmentation patterns in MS/MS data. Therefore, similar fragmentation patterns of related compounds are used as indications of chemical relatedness [31, 32]. In the field of structural prediction of natural products, some useful tools draw great attention. For instance, computational MS-DIAL program can be used to obtain deconvoluted spectra from high-resolution LC–MS data; another computation based MS-FINDER program can be used for structure elucidation of unknown HR-MS spectra through fragment comparison and MS database searching [18, 33]. GNPS provides a visualization approach to detect sets of spectra from related molecules (molecular networks), even when the spectra themselves do not match any known compounds. Using these tools, some metabolites of co-culture were predicted. For example, Ernest et al. [34] analyzed 9 co-cultures of marine-adapted fungi and phytopathogens by GNPS and annotated 18 molecular clusters, 9 of which were exclusively produced in co-cultures. Several clusters contained compounds that could not be annotated to any known compounds, suggesting that they are putatively newly metabolites. However, as only GNPS was mainly involved in these studies, and due to the limited volume of MS/MS library in GNPS, few structures can be predicted. Most structures of newly biosynthesized compounds have not been elucidated yet. Recently, some researchers have also tried to integrate multiple tools to assist structure elucidation. Lai et al. [35] showcased a combined workflow, including GC–MS metabolome database, MS-DIAL and MS-FINDER, to analyze the volatile organic compounds, and three biomarkers and two propofol derivatives were annotated successfully in over 110, 000 biological samples. To our best knowledge, there is still a lack of integrated, effective and accurate strategy to reveal the changes of metabolite profile and characteristics in microorganism following treatment to activate silenced genes, including co-culture.

In this study, MetaboAnalyst, MS-DIAL, MS-FINDER, and GNPS were integrated with the publicly available spectral library to compare the MS/MS data, including common losses of MS and fragmentation similarity, while obtaining the same molecules, analogs, or metabolism families, thereby facilitating structural analysis. Analysis of the co-culture of *A. sydowii* and *B. subtilis* by this new approach revealed 206 features induced in the confrontation zone, and 25 features which occupied 12.1% of the detected features were newly induced by co-culture. Especially, 4 features (**N7**, **N20**, **N9,** and **N6)** were identified as novel compounds. All the 25 newly biosynthesized metabolites were identified by the integrated approach, and the accuracy of the integrated approach was also partially verified by the isolation, purification and spectrum analysis of five newly biosynthesized metabolites with high content. These results suggested that this new approach provided an effective and time-efficient manner to characterize the overall changes of the metabolite profile and to elucidate the structures of metabolites simultaneously. In the integrated approach, **N6** and **N9**, which were derivatives of **N7** with a low content, were detected by GNPS molecular network based on the similar fragment pattern. Their structures were further elucidated with the assistance of MS-DIAL and MS-FINDER programs. These results suggested the new approach was effective to discover trace derivatives of metabolites, and would help to understand the global metabolite profile changes in the co-culture system. Interestingly, the newly biosynthesized features were linearly correlated in the loading plot of the PLS-DA analysis in MetaboAnalyst (Additional file 1: Fig. S2). In the previous co-culture study, a similar phenomenon was presented in the PCA analysis using SIMCA-P software when two fungi, *Trametes versicolor* and *Ganoderma applanatum* were co-cultured (Fig. 1 in reference Xu et al. [36]), although the authors did not describe this linear correlation. These data suggested that using the linear correlation rule of newly biosynthesized metabolites in PLS-DA or PCA analysis, the new biosynthesized metabolites, especially the newly biosynthesized metabolites with low content, might be easily figured out, although the mechanism of this rule still needs to be clarified.

Analysis of the structural features of the part of newly biosynthesized metabolites also revealed their producing microorganism. Sydonic acid (**N2**) has been reported as a typical metabolite of *Aspergillus* sp. [25], suggesting that this compound and its structurally similar compounds **N3**, **N4**, **N6**, **N7,** and **N9** were produced by *A. sydowii* under the inducing stress of *B. subtilis*. Similarly, **N21** has been reported to be produced by the species of *Bacillus* [28], suggesting that this compound and its analogues **N13** and **N20** were supposed to be produced by *B. subtilis*.

The structures of 25 newly biosynthesized metabolites in the co-culture can be categorized into five classes, including macrolides, sesquiterpenes, esters, polyketides, and flavonoids. **N13** and **N20** belonged to macrolides. Macrolide antibiotics have multiple conjugated double bonds, hydroxyl side chain groups, and macrolide skeleton structures. This class of antibiotics has no effect on bacteria, but has an inhibitory effect against fungi. Macrolides can interact with sterols on the membrane of fungal cells, causing the leakage of small molecules and ions in the cell content from the transmembrane pores, eventually leading to the death of fungal cells [37]. For instance, Macrolactin A (**N13**), was reported to display meaningful antifungal activity with MIC values of 0.04–0.3 mM [38]. Compounds **N2**, **N3** and **N4** are classified into sesquiterpenoids, which are widely distributed in nature with anti-bacteria, anti-inflammation and immunoregulatory activities [39]. For example, (7*S*)-(−)-10-Hydroxysydonic acid (**N3**), was reported to display inhibitory activities against *S. aureus* with IC_50_ values ranging from 31.5 to 41.9 μM [40]. (*R*)-(−)-Hydroxy Sydonic acid (**N4**), showed broad spectrum activities against *S. aureus* and *B. cereus*, with MIC less than 25 μM [26]. These results, together with the analysis of the producing microorganisms of these compounds, indicated that in order to exert the antagonistic effect, *A. sydowii* and *B. subtilis* induced the biosynthesis of macrolides and sesquiterpenoids, respectively, to inhibit the growth of the opponent. In addition, inducing the production of sesquiterpenoids by the fungus might play important roles in symbiosis, such as enhancing the immune regulation of bacteria, removing free radicals, enhancing the vitality of bacteria and gaining more nutrients effectively. As the response of antagonistic and symbiosis effects, expression of silent genes was activated and the new metabolites were biosynthesized effectively during the co-culture of *A. sydowii* and *B. subtilis*.

The biological assay indicated that purified newly biosynthesized metabolites showed specific inhibitory activities against PTP1b, SHP1 and CD45. The PTP1b assay data of compounds **N2**–**N7** in this study indicated that the side chains of these compounds influenced the activities. The addition of hydroxyl groups to the side chain lowered the activity significantly. However, the addition of hydroxyl groups to the side chain had no obvious effect on the inhibitory activity against CD45. Further research is still needed to reveal the structure–activity relationship among these compounds, which will help to design new agents for the treatment of diabetes or immunomodulation.

## Conclusions

Co-culture of *A. sydowii* and *B. subtilis* increased the diversity of metabolites. The new integrated approach in this study, which includes MetaboAnalyst, MS-DIAL, MS-FINDER, and GNPS, explored the overall changes of microbial metabolites profile of co-culture, and elucidated the structural information of 25 compounds. Four of the compounds identified are novel. The structures of the other 5 compounds were purified and their NMR data were analyzed to verify the accuracy of the new approach. These data suggest that the new approach is effective and reliable for the rapid identification of metabolites. The biological activities of 7 compounds isolated showed relatively strong inhibitory activity of **N2** to PTP1b, indicating that the co-culture strategy could induce the production of bioactive secondary metabolites, and provide a valuable platform for the discovery of more novel secondary metabolites. The co-culture strategy will also contribute to the revelation of the metabolic mechanisms that can activate silent genes.

## Methods

### General experimental procedures

HPLC analysis was performed with a Waters HPLC system equipped with a 2998 detector and a 1525 pump. Routine detection wavelengths were at 235, 254, 280, and 340 nm. Twenty (20) μL of the samples was injected to a Shimadzu TC-C_18_ column (10 × 250 mm, 5 μm), and the following gradient was used (mobile phase A: 0.2% CH_3_COOH in H_2_O, mobile phase B: acetonitrile): 0–30 min (20–80% B), 30–35 min (80–100% B), 35–40 min (100% B) at 37℃ with a flow rate of 1 mL/min. Compounds were prepared by silica gel column chromatography and an LC3000 semi-preparative HPLC system (Beijing Chuang Xin Tong Heng Science and Technology Co., Ltd). The analytical, semi-preparative and preparative HPLC was performed using an ODS column from Shimadzu Co. (TC-C_18_, 10 × 250 mm, 5 μm), an YMC semi-preparative column (YMC-Pack Pro C_18_ RS, 10 × 250 mm, 5 μm), and an YMC preparative column (YMC-Pack ODS-A, 20 × 250 mm, 10 μm). The NMR data were recorded on a Bruker 500 MHz spectrometer from Bruker Co.

### Microorganisms and co-cultivation experiment

*Aspergillus sydowii* was isolated from a piece of deep-sea mud from Dalian, China. In order to further excavate the secondary metabolites of the strain, *A. sydowii* was co-cultured with *B. subtilis.* The fungal and bacterial strains were activated in potato dextrose agar (PDA) medium (200 g Potato/L, 20 g dextrose/L and 15 g agar/L) for 3 days. Then, each strain was suspended in 2 mL of sterile water. To establish individual pure cultures, 80 μL of bacterial suspension was inoculated into a 90 mm petri dish containing 20 mL of bran medium (100 g bran/L, 20 g dextrose/L, 15 g agar/L). For the co-culture, 80 μL of each bacterial suspension of *A. sydowii* and *B. subtilis* was inoculated approximately 10 mm apart on the PDA medium. The plates were incubated at 28 °C for 12 days.

### Measurement of the metabolome

The extracts were dissolved in 150 μL methanol and centrifuged at 16000×*g* for 10 min. The supernatants were transferred into HPLC autosampler vials and analyzed on a LTQ Orbitrap XL mass spectrometer (Thermo Fisher Scientific, Hemel Hempstead, UK) at a flow rate of 0.6 mL/min. The ESI conditions were as follows: the spray voltage was fixed at 4200 V; the sheath gas pressure was 35 arb; the auxiliary gas pressure was 10 arb and the heater temperature were 320 °C, and the capillary temperature was 300 °C. The mass scanning range was m/z 50–1200 Da in centroid mode with a scan rate of 1.5 spectra/s. The mass detection was performed by an electrospray source functioning in positive and negative ion mode at 15,000 resolving power. The mass measurement was externally calibrated before the experiment. Each full MS scan was followed by data-dependent MS/MS on the three most intense peaks using stepped collision-induced dissociation (35% normalized collision energy, isolation width 2 Da, activation Q 0.250). Twenty (20) μL of the samples was separated by a Shimadzu TC-C_18_ column (10 × 250 mm, 5 μm). The mobile phase A was water with 0.1% acetic acid and the mobile phase B was acetonitrile with 0.1% acetic acid. The elution gradient of reversed-phase liquid chromatography was as follows: 0–10 min, 20% B; 10–30 min, 20–80% B; 30–35 min, 80–85% B; 35–40 min, 100% B; 40–45 min, 25% B; All the samples had three independent biological replicates. The solvent (MeOH) and pure culture were injected under the same conditions as controls.

### Metabolites profile and structure analysis

In order to fully exploit the differences of the metabolite profile in co-culture and pure cultures, MS-DIAL (Version 3.90), the computational approach which helps to rapidly characterize the structure of the metabolites [22], and MetaboAnalyst [41], the web-based tools for comprehensive metabolomic data analysis and interpretation, were integrated. This approach mainly includes: (1) determination of monoisotopic mass of peaks with MS-DIAL. In MS-DIAL, the adduct ion dictionary were defined as: [M+H]^+^, [M+Na]^+^, [M+K]^+^, [M−H_2_O+H]^+^, and [2M+H]^+^ for data from positive ion mode, and [M−H]^−^, [M−H_2_O−H]^−^, [M+HCOOH−H]^−^, [M+CH_3_COOH−H]^−^and [2M−H]^−^ for data from negative ion mode. The monoisotopic mass of each peak was determined when at least two adduct ions matched the adduct ion dictionary. (2) Peak list alignment with MS-DIAL. The MS/MS data were converted to abf format by Analysis Base File Converter, and then subjected to MS-DIAL program to find the peak list alignment. The MS tolerance was set as 0.01 Da, the minimum peak height was set as 1 × 10^7^, and the maximum charge was set to 2. (3) Multivariate analysis of the global metabolites profile with MetaboAnalyst. The aligned data were uploaded to MetaboAnalyst, and the data were first normalized by the sum and auto scaled. The data then were analyzed with PLS-DA to reveal the global profile changes, and the heatmap that could show clustering of the features and visualize the differences between groups was also obtained. (4) Structural identification of the metabolites assisted with MS-DIAL and GNPS. This step mainly included four levels. Level 1: structure annotated on MS-DIAL linked MS/MS databases by the characteristic product ions and neutral losses. The MS/MS public databases mainly include ReSpect, BMDMS-NP, MetaboBASE, Fiehn/Vaniya and natural product library in positive and/or negative manner. Level 2: structure annotated on MS-DIAL linked MS-FINDER program. The metabolite ions were converted into structural information with MS-FINDER. The number of carbon atoms and formula can be determined and the structural formula of all substructures were defined. Compared with the public spectral databases including NIST 14, MassBank, Metlin, ReSpect, and MetaboBase, the compounds with monoisotopic mass error within ± 5 ppm and a structure score higher than 5 were screened for mass spectral peak matching. Then the structures were searched on Reaxys and SciFinder database to confirm whether they were derived from natural products. Finally, the ontology for all substructure forms was defined. Level 3: structure annotation assisted by GNPS. LC–MS/MS data was uploaded to GNPS to create the network between the metabolites. Thus, the features whose structure scores was less than 5 or its fingerprints could not match any compounds in MS/MS database, which might be the novel compounds, might be correlated to the other structures. If any structures in the molecular network can be identified in LC–MS/MS database, the structure of other features in the network can be deduced by comparing the difference between the MS/MS spectrum of unknown and available structures. Otherwise, at least one of the compounds in the network would be separated, purified and its NMR spectrum was analyzed to elucidate the structure of the feature, and then the other structure of the network was deduced accordingly. In GNPS analysis, the LC–MS/MS data were first converted to mzXML format by MS Convert and processed by MZmine 2 [42] and then uploaded to GNPS. The parent mass tolerance was set as 2.0 Da. The ion tolerance was set as 0.5 Da. The maximum connected components value was set as 19 and the minimum cluster size was set as 2. All matches between the network spectra and the library spectra were required to have a score above 0.7 and at least six matched peaks. The output of the molecular networks was visualized using Cytoscape (Version 3.6.1) [43]. Level 4: structure identification by separation, purification and NMR spectrum analysis. The features whose structures could not be determined by Level 1–3 were separated and purified by column chromatography, and analyzed with 1D and 2D NMR spectrum. To verify the veracity of the identification approach, some structures with higher VIP scores in PLS-DA analysis were also separated, purified and analyzed with NMR data.

### Extraction and isolation of the metabolites

After cultured for 12 days, the confrontation zones of co-culture were collected and soaked in ethyl acetate to extract the compounds of interest. The extract was evaporated under reduced pressure, and 30 g of the crude extract was obtained. Then, the crude extract was subjected to a silica gel column and a gradient elution using *N*-hexane/dichloromethane (90:10 → 0:100 over 30 min, 0:100 hold for 10 min) at a flow rate of 12 mL/min. Three fractions, F_1_–F_3_, were obtained from the separation. The fraction F_1_ was further purified using DAISO ODS (20% acetonitrile to 100% acetonitrile over 35 min) at a flow rate of 20 mL/min, followed by preparative HPLC with acetonitrile-H_2_O (30% isocratic) to yield **N1** (20 mg), **N3** (19 mg), **N4** (13 mg) and **N7** (50 mg). The fraction F_2_ was processed in the same manner as F_1_ with acetonitrile-H_2_O (60% isocratic) to yield **N2** (60 mg) and **N13** (21 mg). The fraction F_3_ from the separation was further purified by a YMC preparative column and a YMC semi-preparative column at 3 mL/min with acetonitrile-H_2_O (75% isocratic) to yield **N20** (8 mg).

### Computational details

The theoretical calculations of compound **N7** were performed using Gaussian 09. Firstly, the conformations at B3LYP/6-31G (d) level were optimized in MeOH and the theoretical of ECD was determined using Time Dependent Density Functional Theory (TDDFT) at B3LYP/6-31G (d, p) level in MeOH. Secondly, the ECD spectra was simulated using Gaussian function with band width σ = 0.30 eV. Finally, the ECD spectra of compound **N7** was obtained by weighing the Boltzmann distribution rate of each geometric conformation.

### Protein tyrosine phosphatase 1b inhibitory assay

The PTP1b inhibitory activity of the tested compounds was measured at 37 °C using *p*-nitrophenyl phosphate (pNPP) as the substrate. The reaction was performed in a 96-well plate (final volume of 150 μL) and incubated for 30 min in the assay buffer (50 mM citrate (pH 6.0), 0.1 M NaCl, 1 mM EDTA, and 1 mM dithiothreitol) at 37 °C. Subsequently, the reaction was terminated by the addition of 10 M NaOH and the amount of *p*-nitrophenyl was determined by measuring the absorbance at 405 nm.

## Supplementary Information

**Additional file 1.** Additional figures, tables and compounds information.

## Data Availability

All data generated or analyzed during this study are included in this published article and its Additional file.

## Abbreviations

NPs: Natural products
*A. sydowii*: *Aspergillus sydowii*
*B. subtilis*: *Bacillus subtilis*
MS: Mass spectrometry
GNPS: Global Natural Product Social Molecular Network
PTPs: Protein tyrosine phosphatase
HCA: Hierarchical clustering analysis
VIP: Variable importance in projection
CD45: Leukocyte common antigen
PDA: Potato dextrose agar
PLS-DA: Partial least squares discriminant analysis
TDDFT: Time Dependent Density Functional Theory
