# Supplementary material for: Inducing secondary metabolite production of Aspergillus sydowii through microbial co-culture with Bacillus subtilis
Source: Microb Cell Fact. 2021 Feb 12;20:42. doi: 10.1186/s12934-021-01527-0 (PMC7881642; doi:10.1186/s12934-021-01527-0)
Supplement: Supplementary file 1 — Additional file 1. Additional figures, tables and compounds information. [file 12934_2021_1527_MOESM1_ESM.docx]

**Additional file 1**

**Inducing secondary metabolite production of *Aspergillus sydowii* through microbial co-culture with *Bacillus subtilis***

Yu Sun^1^, Wen-Cai Liu^5^, Xuan Shi^1^, Hai-Zhou Zheng^2^, Zhi-Hui Zheng^2^, Xin-Hua Lu^2^, Yan Xing^1^, Kai Ji^3, 4^, Mei Liu^3, 4^ *, Yue-Sheng Dong^1,^ *

^1^ School of Bioengineering, Dalian University of Technology, Dalian, 116024, Liaoning, China

^2^ New Drug Research and Development Center, North China Pharmaceutical Group Corporation and National Microbial Medicine Engineering and Research Center, Shijiazhuang 050015, Hebei, China

^3^ CAS Key Laboratory of Microbial Physiological and Metabolic Engineering, Institute of Microbiology, Chinese Academy of Sciences, Beijing 100101, China.

^4^ University of Chinese Academy of Sciences, Beijing, 101408, China

^5^ Shandong New Time Pharmaceutical Co., Ltd, Shandong, 255000, China

***Corresponding author:**

Yue-Sheng Dong Ph. D, Associate Professor

Tel./Fax: +86-411-84706344

E-mail: [yshdong@dlut.edu.cn](mailto:yshdong@dlut.edu.cn)

Mei Liu Ph. D, Associate Professor

Tel./Fax: 10-64806978

E-mail: [lium@im.ac.cn](mailto:lium@im.ac.cn)

**
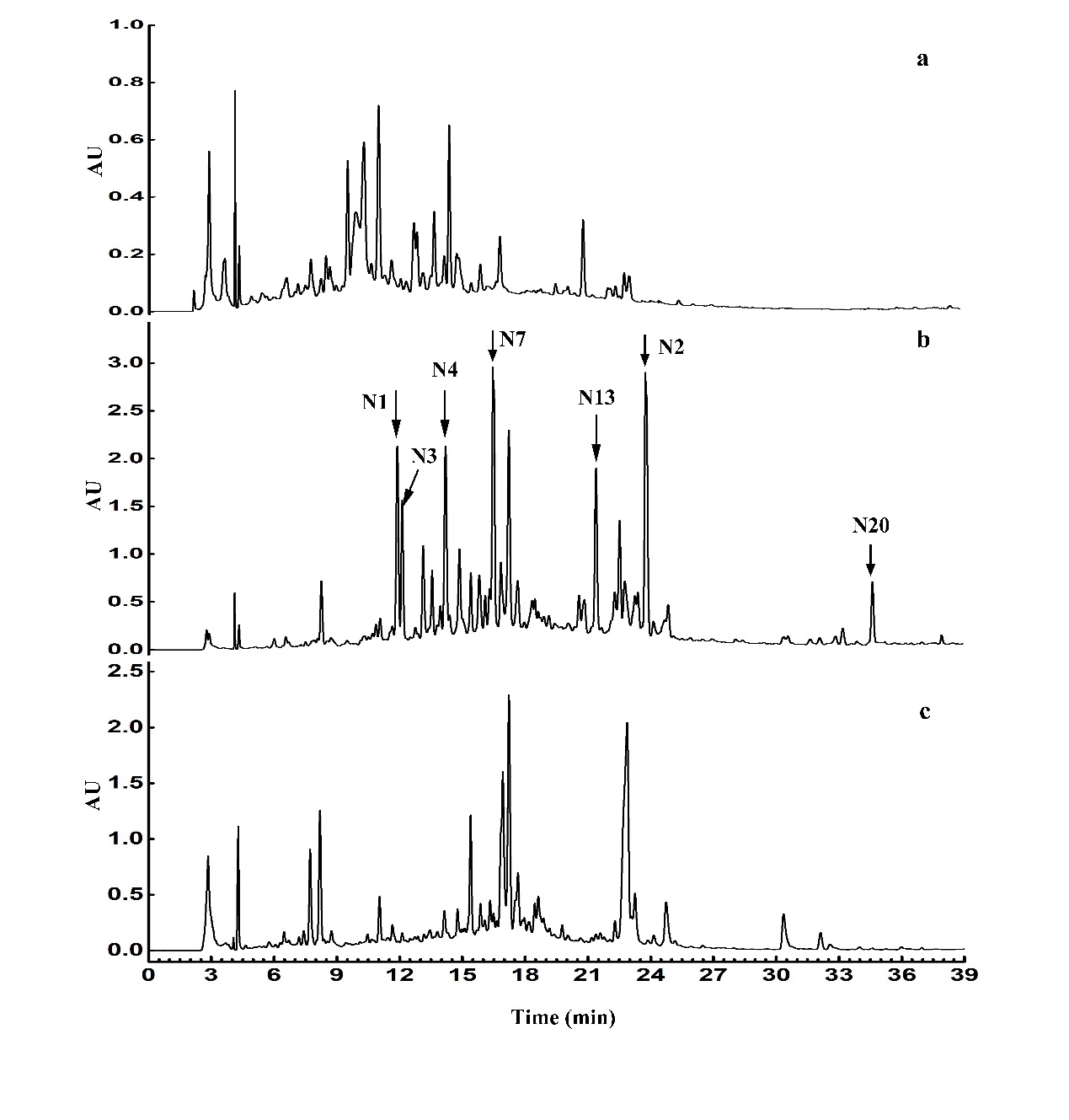
**

Fig. S1 HPLC profiles of extracts of microorganisms cultivated in bran agar plate. (a) Pure culture of *A. sydowii* on day 12. (b) Co-culture of *A. sydowii* and *B. subtilis* on day 12. (c) Pure culture of *B. subtilis* on day 12 by UV absorption at 254nm.


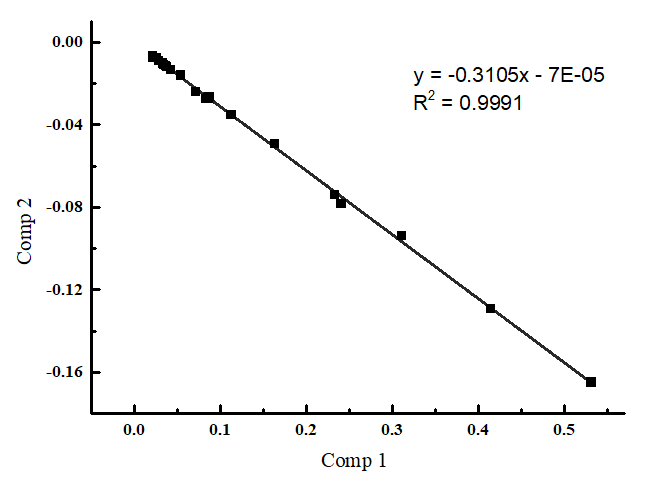


Fig. S2 Features produced by co-culture shown linear relationship


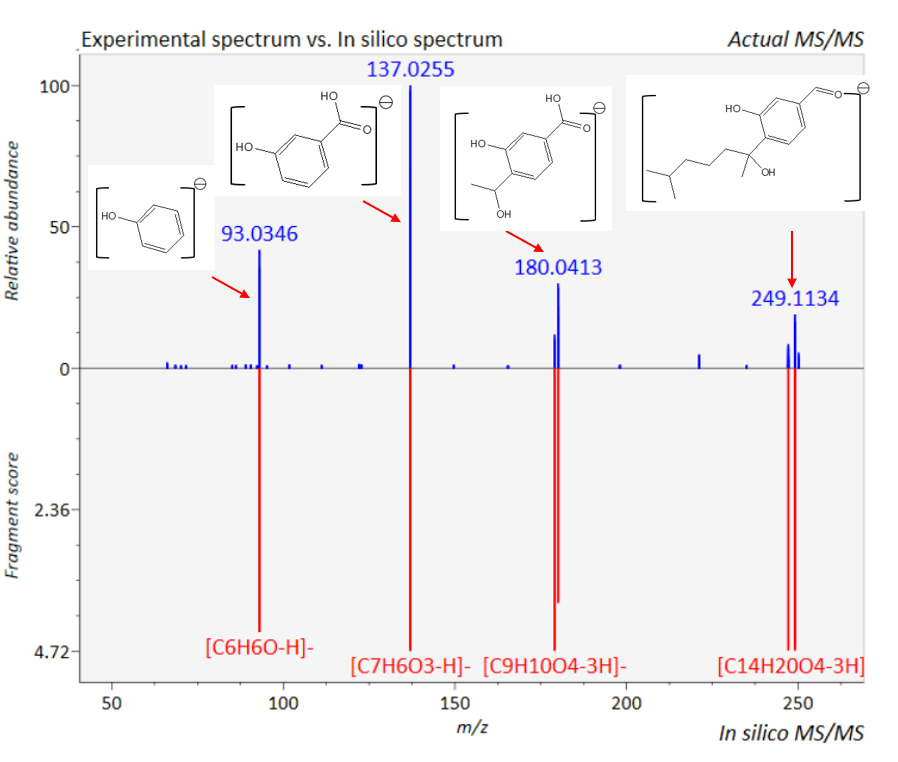


Fig. S3 Mass spectrometry data shows some ion fragments and structures of compound **N2**

**Compounds information**

Orsellinic acid (**N1**)

White crystal; ESI-MS m/z: 167.04 [M-H]^-^ (Calculated for C_8_H_8_O_4_); ^1^H-NMR (500 MHz, CD_3_OD): 2.65 (3H, s), 6.18 (1H, d, *J*=2.1), 6.13 (1H, d, *J*=2.1). ^13^C-NMR (500 MHz, CD_3_OD): 175.2, 167.0, 163.7, 145.3, 112.2, 105.6, 101.6, 24.4. Compared with the data given in reference [[1](#_ENREF_1)], compound **N1** was identified as Orsellinic acid.

Sydonic acid (**N2**)

Colorless needles; ESI-MS m/z: 265.14 [M-H]^-^ (Calculated for C_15_H_22_O_4_); ^1^H-NMR (500 MHz, CD_3_OD): 0.80 (3H, s), 0.82 (3H, s), 1.13 (2H, m), 1.29 (1H, m), 1.34 (1H, m), 1.47 (1H, m), 1.59 (3H, s), 1.77 (1H, ddd, *J*=11.6, 13.7), 1.94 (1H, ddd, *J*=12.2, 13.7), 7.25 (1H, d,7.9), 7.37 (1H, d, 7.6), 7.44 (1H, dd, *J*=,1.6, 7.9). ^13^C-NMR (500 MHz, CD_3_OD): 22.83, 22.89, 22.96, 28.86, 28.95, 40.42, 43.68, 78.0, 118.67, 121.52, 127.74, 131.64, 137.91, 156.96, 169.88. Compared with the data given in reference [[2](#_ENREF_2)], compound **N2** was identified as Sydonic acid.

(7S) - (−)-10-Hydroxysydonic Acid (**N3**)

White amorphous powder; ESI-MS m/z: 281.14 [M-H]^-^ (Calculated for C_15_H_22_O_5_); ^1^H-NMR (500 MHz, CD_3_OD): 0.84 (1H, d, *J*=6.7), 0.86 (1H, d, *J*=6.7), 1.28 (1H, m), 1.52 (1H, m), 1.56 (1H, m), 1.62 (3H, m), 1.81 (1H, ddd, *J*=13.2, 13.1, 4.0), 2.23 (1H, dt, *J*=4.1, 13.1), 3.2 (1H, m), 7.28 (1H, d, *J*=7.4), 7.37 (1H, d, *J*=7.4), 7.44 (1H, s). ^13^C-NMR (500 MHz, CD_3_OD): 17.92, 19.36, 29.1, 29.58, 34.65, 39.96, 77.83, 77.84, 118.67, 121.56, 127.84, 131.72, 137.69, 156.93, 169.92. Compared with the data given in reference [[3](#_ENREF_3)], compound **N3** was identified as (7S) - (-)-10-Hydroxysydonic Acid.

(R)-(-)-Hydroxysydonic acid (**N4**)

Colorless oil; ESI-MS m/z: 281.14 [M-H]^-^ (Calculated for C_15_H_22_O_5_); ^1^H-NMR (500 MHz, CD_3_OD): 1.10 (3H, s), 1.11 (3H, s), 1.27 (1H, t, *J*=7.5, 7.0), 1.37 (1H, m), 1.39 (2H, m), 1.61 (3H, s), 1.97 (2H, m), 7.29 (1H, d, *J*=8.5), 7.37 (1H, d, *J*=2.0), 7.45 (1H, dd, *J*=8.5, 2.0). ^13^C-NMR (500 MHz, CD_3_OD): 170, 156.88, 137.89, 131.8, 127.76, 121.55, 118.65, 77.9, 71.44, 45.03, 43.99, 29.17, 29.07, 28.85, 19.99. Compared with the data given in reference [[4](#_ENREF_4)], compound **N4** was identified as (R) - (-)-Hydroxysydonic acid.

Macrolactin A (**N13**)

White powder; ESI-MS (m/z): 425.23 (M + Na) ^+^, ^1^H-NMR (500 MHz, CD_3_OD): 7.25 (1H, dd, *J* = 11.4, 14.6), 6.64 (1H, m), 6.25 (1H, dd, m), 6.19 (1H, m), 6.04 (1H, m), 5.92 (1H, dd, *J* = 10.9, 11.2 Hz), 5.77 (1H, m), 5.65 (2H, m), 5.59 (1H, d, *J* = 11.0 Hz), 5.52 (1H, dd, *J* = 4.5, 14.6 Hz), 5.43 (1H, m), 5.02 (1H, m), 4.32 (1H, m), 4.24 (1H, m), 3.86 (1H, m), 2.70 (1H, m), 2.45 (1H, m), 2.35 (1H, m), 2.25 (1H, m), 1.70 (1H, m), 1.64 (2H, m), 1.55 (1H, m), 1.37 (1H, m), 1.26 (2H, m), 1.15 (1H, m), 0.96 (3H, d, *J* = 6.3 Hz). ^13^C NMR (CD_3_OD, 500 MHz): 167.98, 144.91, 142.12, 137.54, 135.20, 135.10, 131.68, 131.37, 131.21, 130.24, 128.39, 125.95, 118.01, 72.30, 72.21, 69.84, 69.26, 43.90, 42.82, 36.49, 36.01, 32.96, 25.65, 20.13. Compared with the data given in reference [[5](#_ENREF_5)], compound **N13** was identified as Macrolactin A.

Macrolactin U’ (**N20**)

Pale-yellow cream solid; HRESIMS at m/z 503.3134 [M+Na]^+^ (Calculated for C_31_H_44_O_4_); ^1^H NMR（DMSO-*d6* 500 MHz）: 1.10 (3H, d, *J*=7.0, H-29), 1.39 (1H, m, H-14), 1.41 (1H, m, H-22), 1.47 (1H, m, H-22), 1.59 (1H, m, H-14), 1.68 (3H, s, H-31); 1.71 (3H, s, H-30), 1.91 (3H, s, H-28), 2.01 (1H, m, H-23), 2.10 (2H, m, H-13), 2.13 (1H, m, H-23), 2.23 (2H, m, H-20), 2.31 (1H, m, H-4), 2.56 (2H, m, H-6), 3.41 (2H, m, H-21), 4.25 (1H, m, H-5), 4.45 (2H, t, *J*=7.0, H-15), 4.95 (1H, m, H-27), 5.06 (1H, m, H-27), 5.39 (1H, m, H-19), 5.49 (1H, m, H-7), 5.73 (1H, s, H-2), 5.73 (1H, m, H-12), 5.84 (1H, m, H-25), 6.01 (1H, m, H-10), 6.05 (1H, m, H-17), 6.05 (1H, m, H-9), 6.25 (1H, m, H-18), 6.49 (1H, m, H-11), 6.54 (1H, m, H-26), 6.58 (1H, m, H-8); ^13^C NMR (DMSO-*d6*, 125MHz): 162.76 (C-1), 161.96 (C-3), 140.94 (C-16), 139.66 (C-24), 136.69 (C-12), 133.41 (C-26), 130.46 (C-10), 127.02 (C-19), 126.2 (C-8), 125.69 (C-11), 125.08 (C-25), 124.43 (C-18), 121.78 (C-9), 120.85 (C-17), 115.03 (C-2), 115.03 (C-27), 81.4 (C-5), 69.51 (C-21), 67.08 (C-15), 35.66 (C-4), 35.45 (C-23), 35.05 (C-20), 34.71 (C-14), 34.71 (C-22), 30.8 (C-6), 28.81 (C-13), 21.02 (C-28), 18.24 (C-30), 16.51 (C-31), 15.89 (C-29).

**The original NMR, MS spectrum of compound N7**

Fig. S4 Positive ESI-HRMS spectrum of **N7**


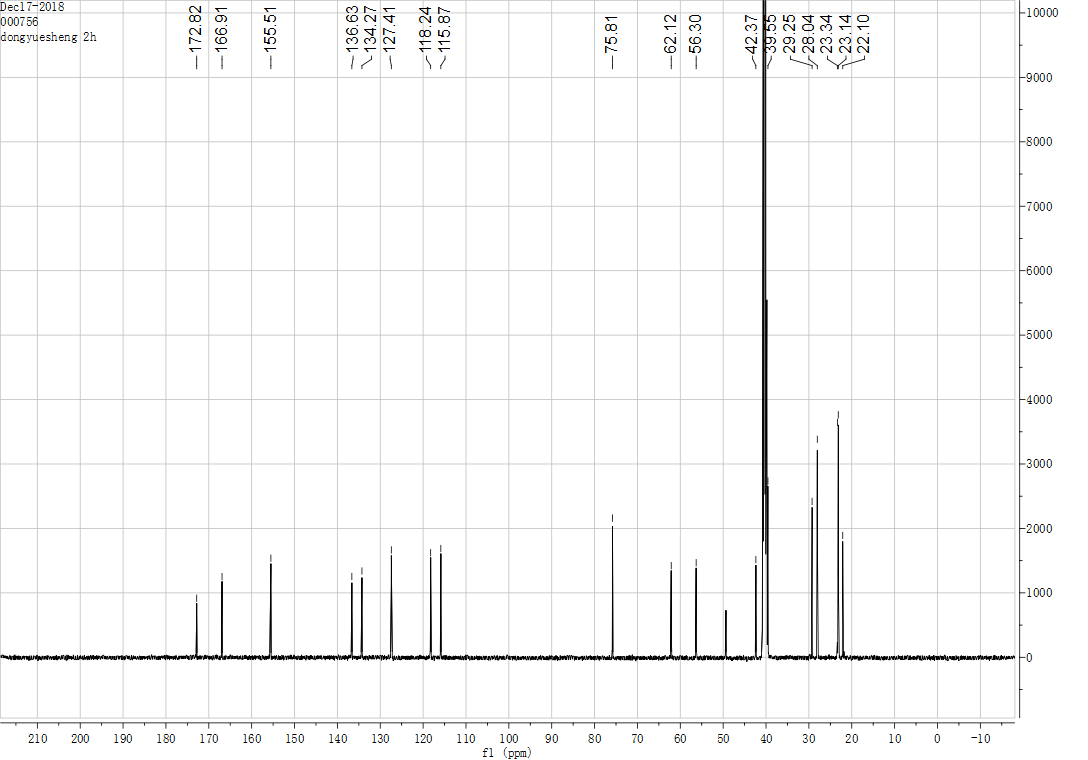


Fig. S5 ^1^H-spectrum of compound **N7** (DMSO-*d6*)


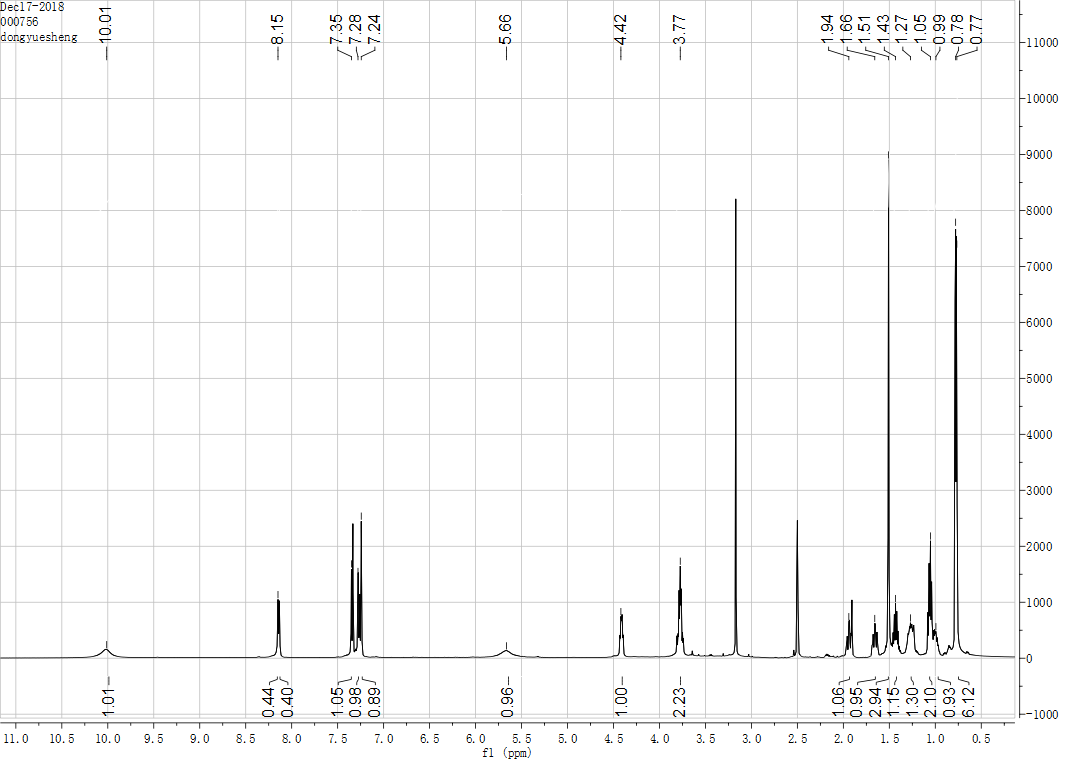


Fig. S6 ^13^C-spectrum of compound **N7** (DMSO-*d6*)


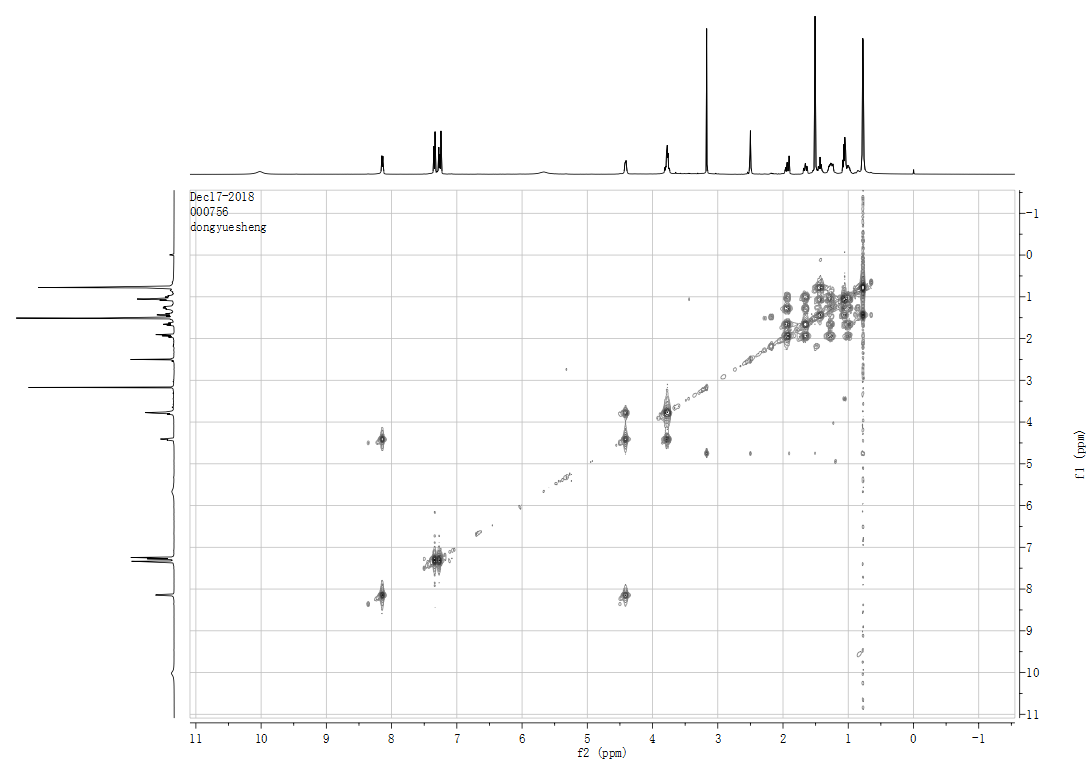


Fig. S7 COSY spectrum of compound **N7** (DMSO-*d6*)


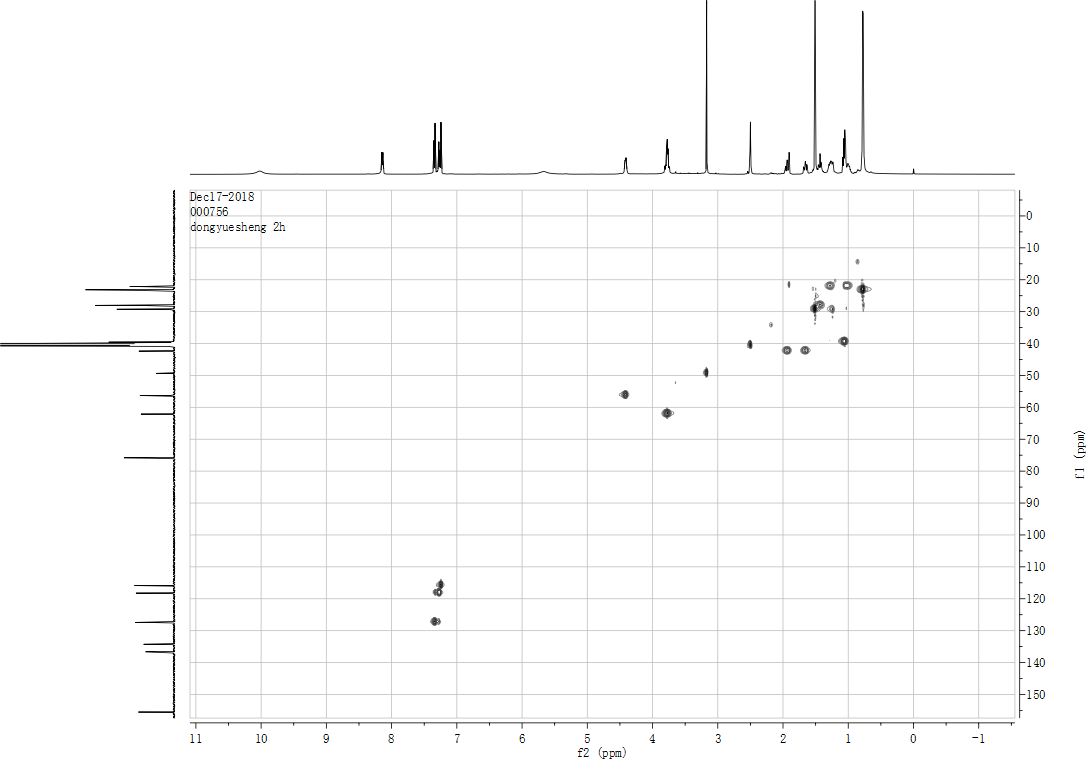


Fig. S8 HMQC spectrum of compound **N7** (DMSO-*d6*)


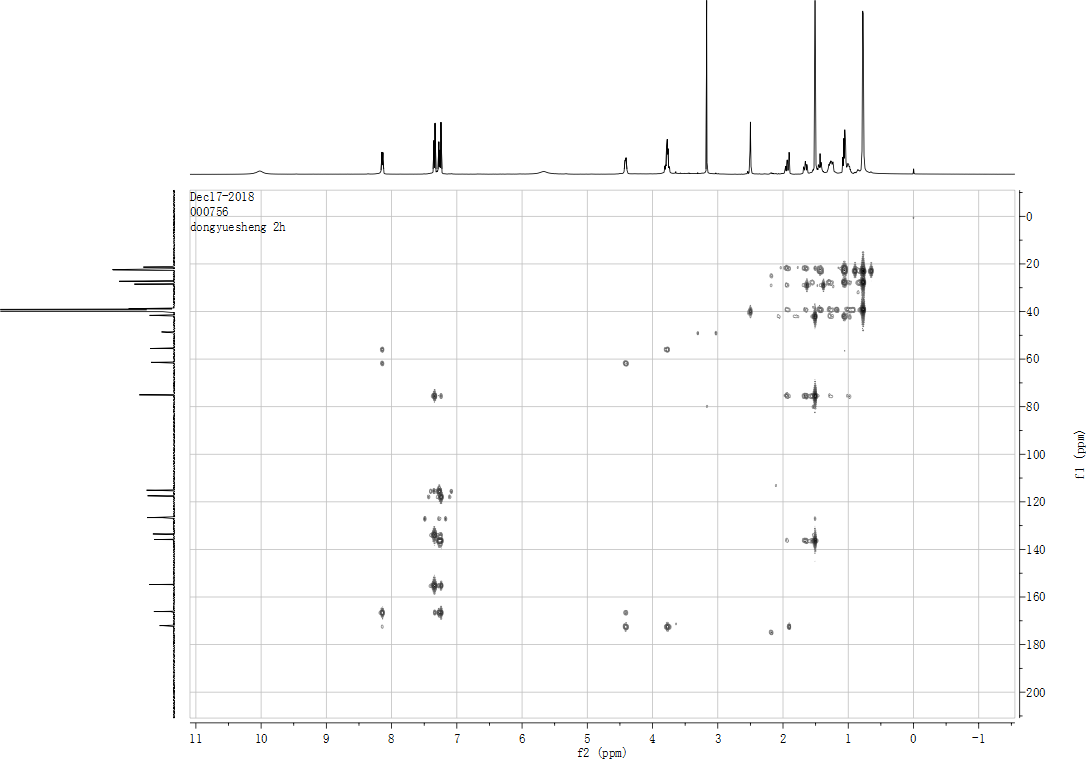


Fig. S9 HMBC spectrum of compound **N7** (DMSO-*d6*)


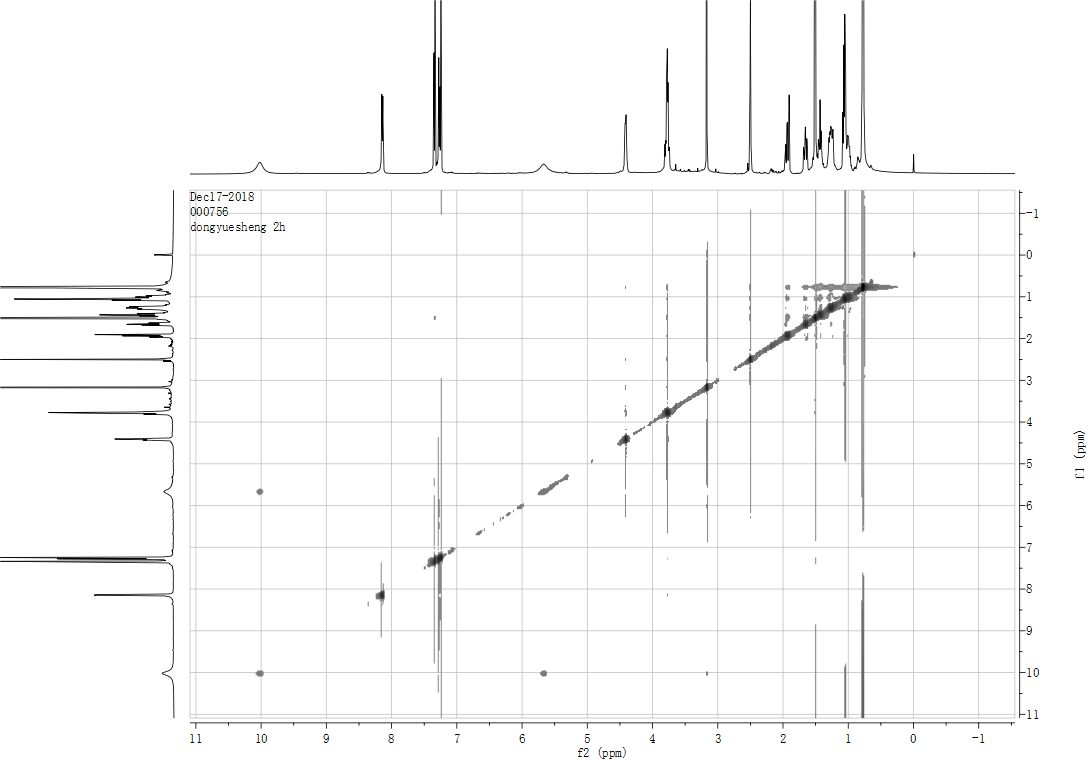


Fig. S10 NOE spectrum of compound **N7** (DMSO-*d6*)

**Computational details of N7**

Stochastic conformational searching was performed by molecular operating environment (MOE). Possible diastereomers [(1’R,8S)-**N7a,** (1’R,8R)-**N7b,** (1’S,8S)-**N7c** and (1’S,8R)-**N7d** for **N7**] were searched, whose relative energies were within 2 kcal/mol. The theoretical calculations of compound **N7** were performed using Gaussian 09 and calculated using GaussView 5.0. The conformations were optimized at B3LYP/6-31G (d) level in MeOH. The theoretical calculation of ECD was performed using Time Dependent Density Functional Theory (TDDFT) at B3LYP/6-31G (d, p) level in MeOH. The ECD spectra were then simulated by using a Gaussian function with band width σ = 0.30 eV. The ECD spectra of compound **N7** were obtained by weighing the Boltzmann distribution rate of each geometric conformation.

Fig. S11 Structure of **N7**a


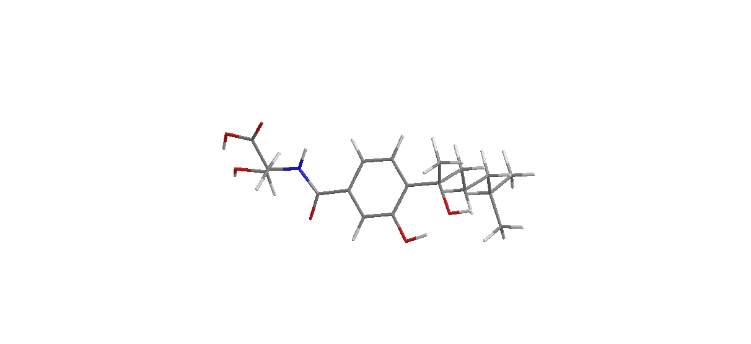

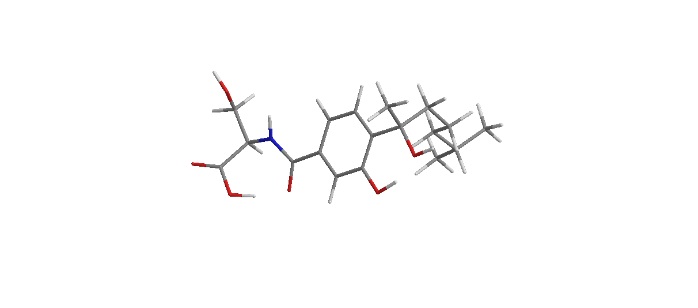

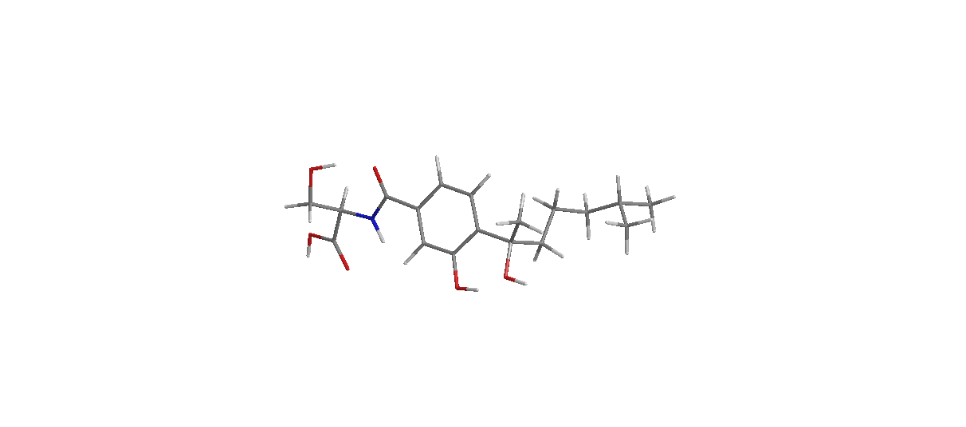


**N7**a-1 **N7**a-2 **N7** a-3

Optimized conformers (≥ 1%) of **N7a** at the B3LYP/6-31+G (d) level with PCM model in MeOH.

Table S1. Important Thermodynamic Parameters and Conformational Analysis of **N7a**

| Conformations | E+ZPE | G | % |
| --- | --- | --- | --- |
| N7a-1 | -1717.53727 | -1207.87822 | 97.10 |
| N7a-2 | -1717.53727 | -1207.87403 | 1.10 |
| N7a-3 | -1717.53719 | -1207.87422 | 1.40 |

E+ZPE: total energy with zero-point energy; G: Gibbs free energy; P: conformational distributions calculated from relative Gibbs free energy.

Table S2. Optimized Cartesian Coordinate of compound **N7**a at B3LYP/6-31G (d, p) level in MeOH Using the PCM Model

| No. | Atom | **N7**a-1 | | | **N7**a-2 | | |
| --- | --- | --- | --- | --- | --- | --- | --- |
|  |  | X | Y | Z | X | Y | Z |
| 1 | C | 0.84148 | -0.86195 | -0.81165 | -0.37394 | 0.08563 | 0.77842 |
| 2 | C | 1.15689 | -0.22788 | 0.39816 | -1.02449 | -0.47636 | -0.32878 |
| 3 | C | -0.40123 | -1.47102 | -0.96175 | 0.92488 | -0.3111 | 1.08334 |
| 4 | C | 0.20844 | -0.20895 | 1.42014 | -0.35265 | -1.42151 | -1.10506 |
| 5 | C | -1.36674 | -1.48263 | 0.0556 | 1.62539 | -1.25669 | 0.32123 |
| 6 | C | -6.14792 | 3.25345 | -1.35296 | 3.43331 | 3.33846 | -1.65493 |
| 7 | C | -1.04046 | -0.82247 | 1.26712 | 0.94652 | -1.82995 | -0.78533 |
| 8 | C | 2.46975 | 0.4502 | 0.65372 | -2.40933 | -0.10186 | -0.73321 |
| 9 | C | -5.28645 | 2.41654 | -0.39514 | 4.42776 | 2.48794 | -0.8497 |
| 10 | C | -5.00092 | 1.02863 | -1.00288 | 3.67792 | 1.40681 | -0.04244 |
| 11 | C | -2.73476 | -2.12906 | -0.21184 | 3.00748 | -1.75929 | 0.7706 |
| 12 | C | 5.84427 | -0.49863 | -0.44007 | -5.6166 | -0.02679 | -0.33303 |
| 13 | C | -3.99777 | 0.1692 | -0.21951 | 4.58928 | 0.35573 | 0.60651 |
| 14 | C | -5.9499 | 2.32953 | 0.98794 | 5.30561 | 3.38869 | 0.03353 |
| 15 | C | 5.08733 | 1.94222 | -0.49246 | -4.86889 | 1.97424 | 1.0783 |
| 16 | C | 4.85429 | 0.54185 | 0.10403 | -4.51818 | 1.03252 | -0.07098 |
| 17 | C | -3.6876 | -1.14894 | -0.94093 | 3.9105 | -0.71784 | 1.4788 |
| 18 | C | -2.61706 | -3.43575 | -1.01411 | 2.79143 | -2.97586 | 1.69336 |
| 19 | H | -2.6521 | -1.32039 | 2.13094 | 2.4379 | -2.8622 | -1.33527 |
| 20 | H | -6.1508 | 3.3306 | 1.38798 | 5.81146 | 4.15218 | -0.56964 |
| 21 | H | -4.2366 | -2.67247 | 0.97213 | 4.45936 | -2.7461 | -0.15735 |
| 22 | H | -5.32162 | 1.8041 | 1.71518 | 6.07947 | 2.82399 | 0.5645 |
| 23 | H | 0.43385 | 0.28676 | 2.35801 | -0.84154 | -1.86591 | -1.96481 |
| 24 | H | -6.90886 | 1.79739 | 0.92665 | 4.69558 | 3.90874 | 0.78441 |
| 25 | H | -6.3241 | 4.25911 | -0.95272 | 2.83143 | 2.7181 | -2.32983 |
| 26 | H | -3.60144 | -3.91375 | -1.06737 | 2.28758 | -2.6734 | 2.61681 |
| 27 | H | 5.01866 | 0.62715 | 1.18803 | -4.43303 | 1.61929 | -0.99491 |
| 28 | H | -7.12718 | 2.7823 | -1.51097 | 3.95254 | 4.0904 | -2.2614 |
| 29 | H | -4.32055 | 2.93152 | -0.27509 | 5.08551 | 1.97207 | -1.56665 |
| 30 | H | -2.27988 | -3.26537 | -2.04032 | 2.17645 | -3.73274 | 1.19627 |
| 31 | H | -1.92253 | -4.12456 | -0.52445 | 3.75355 | -3.42827 | 1.96495 |
| 32 | H | -4.62796 | -1.68775 | -1.13437 | 4.71064 | -1.29457 | 1.96248 |
| 33 | H | -3.25414 | -0.93472 | -1.92636 | 3.35964 | -0.24903 | 2.30185 |
| 34 | H | 4.30397 | 2.61266 | -0.12765 | -5.91364 | 2.28793 | 0.97555 |
| 35 | H | 5.04922 | 1.90532 | -1.5874 | -4.22395 | 2.85982 | 1.01709 |
| 36 | H | 6.67986 | 3.1119 | -0.58825 | -4.65372 | 1.91011 | 3.02206 |
| 37 | H | -0.62799 | -1.94395 | -1.91124 | 1.40537 | 0.13999 | 1.94388 |
| 38 | H | 1.53403 | -0.85855 | -1.64709 | -0.84883 | 0.85128 | 1.3832 |
| 39 | H | -3.07002 | 0.73866 | -0.07626 | 5.16974 | -0.14537 | -0.1782 |
| 40 | H | -4.3894 | -0.04685 | 0.78118 | 5.32015 | 0.85788 | 1.25264 |
| 41 | H | 7.17293 | 0.85299 | -0.25292 | -4.4254 | -0.71472 | -1.68144 |
| 42 | H | 3.45889 | -0.80926 | -0.65233 | -2.95587 | 0.41866 | 1.18979 |
| 43 | H | -5.95088 | 0.48118 | -1.1049 | 3.06862 | 1.90011 | 0.73027 |
| 44 | H | -4.61953 | 1.16812 | -2.02503 | 2.97046 | 0.90606 | -0.71411 |
| 45 | H | -5.66728 | 3.36034 | -2.33296 | 2.74325 | 3.8699 | -0.98602 |
| 46 | N | 3.51033 | 0.06824 | -0.14714 | -3.21209 | 0.43785 | 0.20934 |
| 47 | O | 2.60907 | 1.30894 | 1.52941 | -2.82174 | -0.28745 | -1.89983 |
| 48 | O | -3.28944 | -2.48846 | 1.08661 | 3.70215 | -2.20198 | -0.43155 |
| 49 | O | 6.37777 | 2.37721 | -0.03026 | -4.64884 | 1.26644 | 2.29697 |
| 50 | O | -1.88812 | -0.7281 | 2.3263 | 1.48219 | -2.79798 | -1.57433 |
| 51 | O | 7.12129 | -0.11348 | -0.4838 | -5.32786 | -0.91162 | -1.29233 |
| 52 | O | 5.49934 | -1.60811 | -0.79737 | -6.68517 | -0.04188 | 0.24167 |

| No. | Atom | **N7**a-3 | | |
| --- | --- | --- | --- | --- |
|  |  | X | Y | Z |
| 1 | C | 0.56746 | -1.38429 | -1.38465 |
| 2 | C | 1.41677 | -0.87111 | -0.39816 |
| 3 | C | -0.76369 | -1.65749 | -1.07834 |
| 4 | C | 0.91996 | -0.67278 | 0.8941 |
| 5 | C | -1.29296 | -1.44725 | 0.20117 |
| 6 | C | -6.69271 | 0.9547 | -0.10171 |
| 7 | C | -0.4144 | -0.95868 | 1.19946 |
| 8 | C | 2.83965 | -0.58572 | -0.77122 |
| 9 | C | -5.8106 | 1.91877 | -0.90882 |
| 10 | C | -4.32173 | 1.87897 | -0.50404 |
| 11 | C | -2.77692 | -1.66984 | 0.51072 |
| 12 | C | 5.09175 | 1.94672 | 0.70018 |
| 13 | C | -3.60287 | 0.51908 | -0.67818 |
| 14 | C | -6.34914 | 3.35381 | -0.80396 |
| 15 | C | 5.95397 | -0.28636 | -0.20419 |
| 16 | C | 4.84528 | 0.80144 | -0.27653 |
| 17 | C | -3.53156 | -0.31676 | 0.60985 |
| 18 | C | -3.46892 | -2.64215 | -0.44998 |
| 19 | H | -1.65233 | -1.24302 | 2.60183 |
| 20 | H | -6.32109 | 3.7074 | 0.23529 |
| 21 | H | -3.71681 | -2.25934 | 2.15914 |
| 22 | H | -7.38906 | 3.4131 | -1.14682 |
| 23 | H | 1.55245 | -0.33488 | 1.70913 |
| 24 | H | -5.75486 | 4.04949 | -1.40865 |
| 25 | H | -7.74219 | 1.04055 | -0.4087 |
| 26 | H | -3.53638 | -2.25025 | -1.46802 |
| 27 | H | 4.89216 | 1.22466 | -1.28983 |
| 28 | H | -6.64122 | 1.17933 | 0.97198 |
| 29 | H | -5.868 | 1.61654 | -1.96622 |
| 30 | H | -2.93721 | -3.59775 | -0.47678 |
| 31 | H | -4.49132 | -2.8251 | -0.10048 |
| 32 | H | -3.04491 | 0.27888 | 1.39197 |
| 33 | H | -4.54622 | -0.5362 | 0.96923 |
| 34 | H | 5.73063 | -0.94177 | 0.6522 |
| 35 | H | 6.91608 | 0.19859 | -0.02056 |
| 36 | H | 5.16558 | -1.2399 | -1.68301 |
| 37 | H | -1.40608 | -2.04 | -1.86373 |
| 38 | H | 0.95275 | -1.5585 | -2.38272 |
| 39 | H | -4.09766 | -0.05064 | -1.47561 |
| 40 | H | -2.57917 | 0.69989 | -1.02634 |
| 41 | H | 6.37421 | 3.25663 | 1.13855 |
| 42 | H | 3.00708 | 0.8169 | 0.72263 |
| 43 | H | -3.79826 | 2.62932 | -1.1109 |
| 44 | H | -4.2233 | 2.21736 | 0.53855 |
| 45 | H | -6.39573 | -0.09021 | -0.24226 |
| 46 | N | 3.50336 | 0.30481 | 0.00202 |
| 47 | O | 3.36667 | -1.14517 | -1.74961 |
| 48 | O | -2.79978 | -2.28879 | 1.83631 |
| 49 | O | 6.08054 | -1.002 | -1.41427 |
| 50 | O | -0.80999 | -0.74122 | 2.4838 |
| 51 | O | 6.27774 | 2.53302 | 0.48894 |
| 52 | O | 4.30621 | 2.29651 | 1.55878 |

Fig. S12 Structure of **N7**b


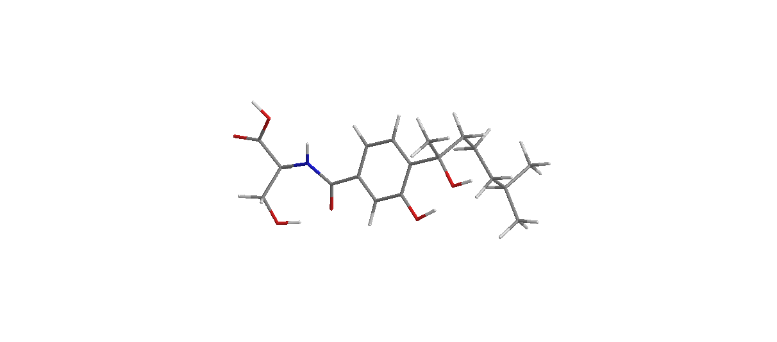

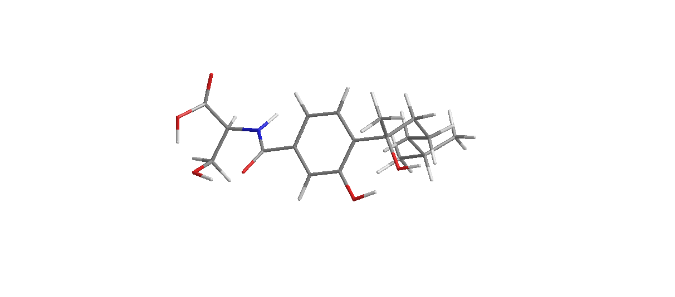

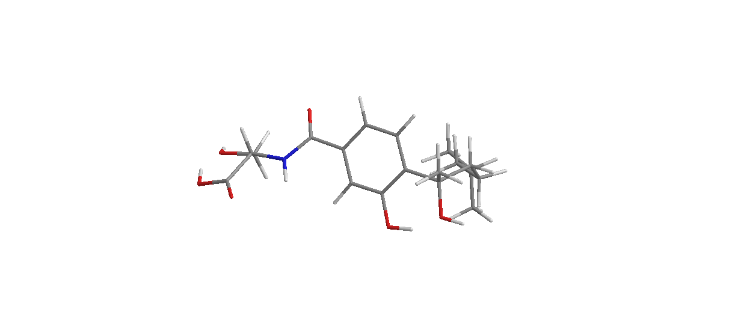


**N7**b-1 **N7**b-2 **N7**b-3


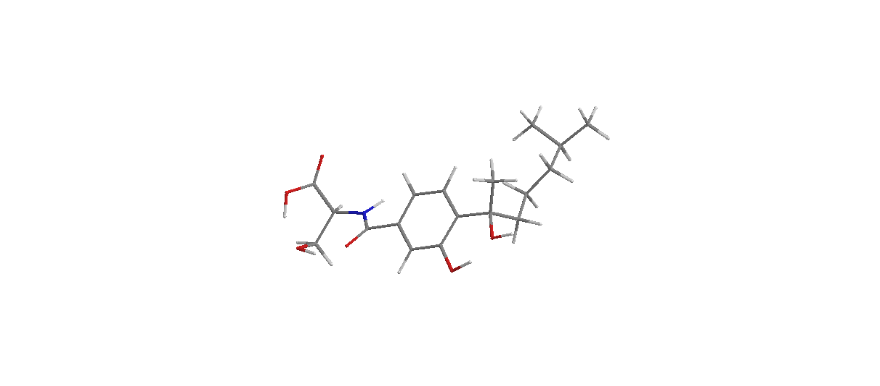


**N7**b-4

Optimized conformers (≥ 1%) of **N7b** at the B3LYP/6-31+G (d) level with PCM model in MeOH.

Table S3. Important Thermodynamic Parameters and Conformational Analysis of **N7b**

| Conformations | E+ZPE | G | % |
| --- | --- | --- | --- |
| N7b-1 | -1716.85067 | -1207.87289 | 5.90 |
| N7b-2 | -1716.85072 | -1207.87441 | 29.50 |
| N7b-3 | -1716.85072 | -1207.87389 | 17.00 |
| N7b-4 | -1716.85071 | -1207.87484 | 46.60 |

E+ZPE: total energy with zero-point energy; G: Gibbs free energy; P: conformational distributions calculated from relative Gibbs free energy.

Table S4. Optimized Cartesian Coordinate of compound **N7**b at B3LYP/6-31G (d, p) level in MeOH Using the PCM Model

| No. | Atom | **N7**b-1 | | | **N7**b-2 | | |
| --- | --- | --- | --- | --- | --- | --- | --- |
|  |  | X | Y | Z | X | Y | Z |
| 1 | C | -0.87001 | 1.11846 | -0.46998 | -0.35573 | -0.26207 | 1.02231 |
| 2 | C | -1.16472 | 0.00457 | 0.32953 | -1.03354 | -0.63203 | -0.1476 |
| 3 | C | 0.38101 | 1.71783 | -0.37022 | 0.98147 | -0.61563 | 1.18056 |
| 4 | C | -0.18765 | -0.48478 | 1.19476 | -0.35771 | -1.37315 | -1.1173 |
| 5 | C | 1.37763 | 1.25832 | 0.50564 | 1.69675 | -1.32655 | 0.20701 |
| 6 | C | 5.85696 | -1.91093 | -2.22129 | 3.07894 | 3.55084 | -1.27171 |
| 7 | C | 1.07134 | 0.12152 | 1.29451 | 0.98515 | -1.73075 | -0.95238 |
| 8 | C | -2.47885 | -0.71124 | 0.28389 | -2.46738 | -0.29095 | -0.40511 |
| 9 | C | 4.95912 | -2.00429 | -0.97772 | 4.16413 | 2.71173 | -0.57938 |
| 10 | C | 4.62732 | -0.62147 | -0.37727 | 3.53888 | 1.47212 | 0.09455 |
| 11 | C | 2.73407 | 1.98187 | 0.50555 | 3.14007 | -1.78485 | 0.4769 |
| 12 | C | -5.7497 | 0.47308 | -1.12437 | -5.11393 | 0.49652 | 1.31173 |
| 13 | C | 3.7194 | 0.25145 | -1.2591 | 4.56244 | 0.43929 | 0.58687 |
| 14 | C | 5.60889 | -2.9067 | 0.08243 | 4.96574 | 3.58157 | 0.40156 |
| 15 | C | -5.60425 | -1.12015 | 0.8297 | -5.02005 | 1.38244 | -1.14206 |
| 16 | C | -4.86492 | -0.54681 | -0.41167 | -4.32775 | 1.2954 | 0.23197 |
| 17 | C | 3.51878 | 1.70865 | -0.80367 | 4.00967 | -0.80587 | 1.30655 |
| 18 | C | 2.55864 | 3.49915 | 0.70252 | 3.08175 | -3.15826 | 1.17478 |
| 19 | H | 2.74215 | 0.11551 | 2.18907 | 2.50199 | -2.51949 | -1.77034 |
| 20 | H | 4.95565 | -3.03261 | 0.95417 | 4.31809 | 3.95689 | 1.20538 |
| 21 | H | 4.43543 | 1.63822 | 1.48058 | 4.58521 | -2.47427 | -0.69722 |
| 22 | H | 6.55635 | -2.47712 | 0.43442 | 5.39697 | 4.45056 | -0.1099 |
| 23 | H | -0.39355 | -1.3532 | 1.81073 | -0.87132 | -1.68693 | -2.01927 |
| 24 | H | 5.82643 | -3.90229 | -0.32261 | 5.79068 | 3.03119 | 0.86674 |
| 25 | H | 6.8213 | -1.44972 | -1.96927 | 2.35111 | 3.92945 | -0.54164 |
| 26 | H | 1.98991 | 3.96245 | -0.10814 | 4.09179 | -3.5687 | 1.29948 |
| 27 | H | -4.75016 | -1.3847 | -1.11271 | -4.27415 | 2.31446 | 0.62339 |
| 28 | H | 6.06233 | -2.90791 | -2.62919 | 3.51349 | 4.41667 | -1.78587 |
| 29 | H | 4.00899 | -2.47255 | -1.27893 | 4.85656 | 2.35316 | -1.35732 |
| 30 | H | 3.54354 | 3.97811 | 0.73848 | 2.48702 | -3.86414 | 0.58637 |
| 31 | H | 2.04314 | 3.69497 | 1.64738 | 2.62846 | -3.06807 | 2.16703 |
| 32 | H | 2.99719 | 2.24726 | -1.60418 | 3.47234 | -0.51376 | 2.21526 |
| 33 | H | 4.49837 | 2.20194 | -0.70664 | 4.87273 | -1.38748 | 1.65841 |
| 34 | H | -6.6725 | -1.16129 | 0.60209 | -4.32878 | 1.82976 | -1.86538 |
| 35 | H | -5.45537 | -0.4229 | 1.66956 | -5.88934 | 2.03868 | -1.04789 |
| 36 | H | -4.2133 | -2.42947 | 1.09738 | -4.71206 | -0.45537 | -1.65611 |
| 37 | H | 0.59248 | 2.56836 | -1.01051 | 1.47531 | -0.33206 | 2.1029 |
| 38 | H | -1.58334 | 1.49976 | -1.1938 | -0.86142 | 0.25704 | 1.83042 |
| 39 | H | 4.14051 | 0.30213 | -2.2708 | 5.16895 | 0.11022 | -0.26593 |
| 40 | H | 2.7409 | -0.23546 | -1.36456 | 5.25712 | 0.91831 | 1.28828 |
| 41 | H | -5.7723 | 2.23902 | -1.80301 | -6.0889 | -0.32197 | -0.11378 |
| 42 | H | -3.42747 | 1.0052 | -0.29397 | -2.27974 | 1.36793 | 0.7493 |
| 43 | H | 4.14516 | -0.77728 | 0.59379 | 2.90532 | 1.80226 | 0.9317 |
| 44 | H | 5.57078 | -0.08922 | -0.17548 | 2.86516 | 0.99277 | -0.62575 |
| 45 | H | 5.40144 | -1.3166 | -3.0207 | 2.52931 | 2.95939 | -2.01388 |
| 46 | N | -3.54738 | 0.01669 | -0.12172 | -2.93336 | 0.83431 | 0.19381 |
| 47 | O | -2.56925 | -1.90742 | 0.61289 | -3.16339 | -1.00034 | -1.15455 |
| 48 | O | 3.48966 | 1.51321 | 1.65774 | 3.77692 | -1.94977 | -0.82323 |
| 49 | O | -5.19568 | -2.43429 | 1.14137 | -5.51548 | 0.1267 | -1.61658 |
| 50 | O | 1.93802 | -0.45499 | 2.16982 | 1.5309 | -2.48223 | -1.94409 |
| 51 | O | -5.13743 | 1.65166 | -1.34859 | -6.08084 | -0.31998 | 0.8879 |
| 52 | O | -6.89164 | 0.24076 | -1.45578 | -4.84832 | 0.6162 | 2.48975 |

| No. | Atom | **N7**b-3 | | | **N7**b-4 | | |
| --- | --- | --- | --- | --- | --- | --- | --- |
|  |  | X | Y | Z | X | Y | Z |
| 1 | C | 0.04888 | 0.02484 | 1.60727 | -0.54502 | 0.11342 | 0.85775 |
| 2 | C | -0.88052 | -0.12151 | 0.57088 | -1.31297 | -0.65137 | -0.02986 |
| 3 | C | 1.32382 | -0.51435 | 1.47277 | 0.77905 | -0.24578 | 1.10761 |
| 4 | C | -0.49918 | -0.79873 | -0.58919 | -0.74537 | -1.77947 | -0.62758 |
| 5 | C | 1.73864 | -1.19412 | 0.31772 | 1.38299 | -1.3489 | 0.49269 |
| 6 | C | 5.19921 | 2.48003 | -1.67114 | 4.11287 | 3.31432 | -0.14026 |
| 7 | C | 0.78486 | -1.34412 | -0.72051 | 0.58365 | -2.13018 | -0.38022 |
| 8 | C | -2.24577 | 0.46755 | 0.75998 | -2.74419 | -0.34511 | -0.344 |
| 9 | C | 4.69004 | 2.6103 | -0.22701 | 5.19844 | 2.4245 | -0.76606 |
| 10 | C | 3.96716 | 1.33868 | 0.2639 | 5.0574 | 0.94405 | -0.3594 |
| 11 | C | 3.11311 | -1.88286 | 0.26611 | 2.86804 | -1.67373 | 0.69057 |
| 12 | C | -5.52309 | -0.78006 | -0.33469 | -5.17188 | 1.35408 | 1.03467 |
| 13 | C | 4.8626 | 0.09388 | 0.34364 | 3.77889 | 0.2587 | -0.87029 |
| 14 | C | 3.77133 | 3.83419 | -0.09134 | 6.59626 | 2.94969 | -0.40504 |
| 15 | C | -4.94101 | 1.70321 | -0.49125 | -5.30163 | 1.01705 | -1.54821 |
| 16 | C | -4.61937 | 0.35233 | 0.17413 | -4.47139 | 1.50611 | -0.34591 |
| 17 | C | 4.26017 | -1.15568 | 1.01322 | 3.68766 | -1.25359 | -0.5615 |
| 18 | C | 2.94741 | -3.31108 | 0.82279 | 3.44995 | -1.11291 | 1.99043 |
| 19 | H | 1.99925 | -2.20753 | -1.89194 | 1.87914 | -3.49986 | -0.5381 |
| 20 | H | 2.8775 | 3.72286 | -0.71943 | 7.38316 | 2.3538 | -0.88297 |
| 21 | H | 4.20551 | -2.62219 | -1.21866 | 3.85995 | -3.39485 | 0.70817 |
| 22 | H | 4.28347 | 4.75289 | -0.40206 | 6.76048 | 2.91062 | 0.68006 |
| 23 | H | -1.17071 | -0.91013 | -1.43484 | -1.33558 | -2.39836 | -1.29412 |
| 24 | H | 3.43575 | 3.96926 | 0.94406 | 6.72301 | 3.99166 | -0.72253 |
| 25 | H | 4.36545 | 2.29232 | -2.36108 | 4.1622 | 3.27506 | 0.95628 |
| 26 | H | 2.67334 | -3.28084 | 1.88207 | 4.48006 | -1.46719 | 2.10762 |
| 27 | H | -4.81035 | 0.47545 | 1.25015 | -4.33404 | 2.58262 | -0.47413 |
| 28 | H | 5.69682 | 3.40228 | -1.99462 | 3.10437 | 3.01003 | -0.44065 |
| 29 | H | 5.56264 | 2.77127 | 0.42554 | 5.09136 | 2.48003 | -1.86079 |
| 30 | H | 3.88582 | -3.87217 | 0.73124 | 2.86613 | -1.45666 | 2.84947 |
| 31 | H | 2.16335 | -3.84732 | 0.27885 | 3.47581 | -0.02066 | 1.99857 |
| 32 | H | 3.94872 | -0.92844 | 2.03874 | 3.24456 | -1.77535 | -1.41643 |
| 33 | H | 5.06888 | -1.8924 | 1.11484 | 4.70248 | -1.66192 | -0.44282 |
| 34 | H | -4.88317 | 1.6179 | -1.5826 | -4.67904 | 1.0386 | -2.4499 |
| 35 | H | -4.21323 | 2.44405 | -0.14848 | -6.13506 | 1.70997 | -1.69102 |
| 36 | H | -6.61235 | 2.74875 | -0.66242 | -5.104 | -0.87503 | -1.21268 |
| 37 | H | 2.01432 | -0.40402 | 2.30138 | 1.34973 | 0.3571 | 1.80509 |
| 38 | H | -0.23807 | 0.55118 | 2.51062 | -0.97046 | 0.95746 | 1.3915 |
| 39 | H | 5.20105 | -0.18525 | -0.65962 | 2.88831 | 0.7669 | -0.48194 |
| 40 | H | 5.76766 | 0.35197 | 0.91199 | 3.73971 | 0.38784 | -1.95975 |
| 41 | H | -6.94673 | 0.48013 | -0.22638 | -6.28963 | 0.02796 | 0.23115 |
| 42 | H | -3.11669 | -0.93081 | -0.48949 | -2.38859 | 1.62557 | -0.0219 |
| 43 | H | 3.54651 | 1.54196 | 1.2589 | 5.92329 | 0.39081 | -0.75113 |
| 44 | H | 3.10994 | 1.14328 | -0.3944 | 5.12254 | 0.87134 | 0.73544 |
| 45 | H | 5.91719 | 1.66053 | -1.78368 | 4.2451 | 4.36055 | -0.44098 |
| 46 | N | -3.24042 | -0.0339 | -0.03319 | -3.10873 | 0.96005 | -0.26574 |
| 47 | O | -2.46514 | 1.36055 | 1.58388 | -3.52374 | -1.25383 | -0.68317 |
| 48 | O | 3.48887 | -1.97052 | -1.13933 | 2.92708 | -3.13159 | 0.78765 |
| 49 | O | -6.26661 | 2.06509 | -0.06627 | -5.8829 | -0.27631 | -1.35845 |
| 50 | O | 1.031 | -2.01635 | -1.87669 | 1.05906 | -3.23334 | -1.01953 |
| 51 | O | -6.82343 | -0.48967 | -0.41191 | -6.18011 | 0.48342 | 1.11683 |
| 52 | O | -5.09452 | -1.87693 | -0.63593 | -4.80195 | 1.99745 | 1.99494 |

Fig. S13 Structure of **N7**c


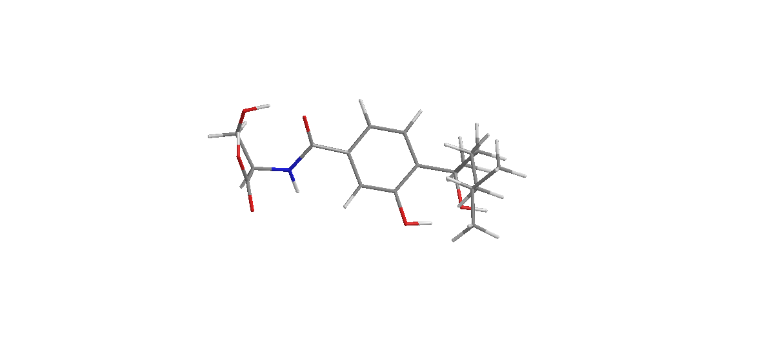

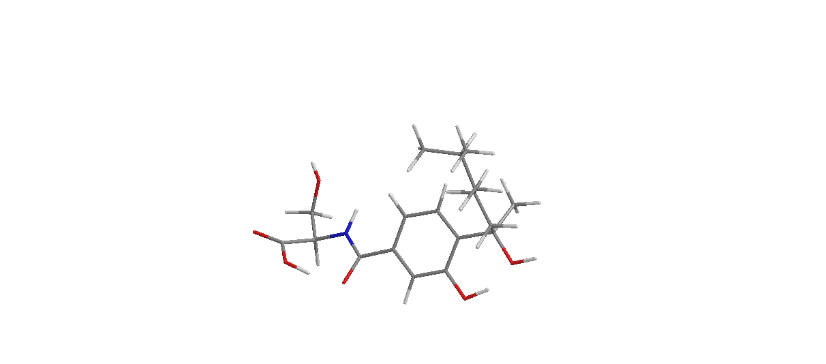

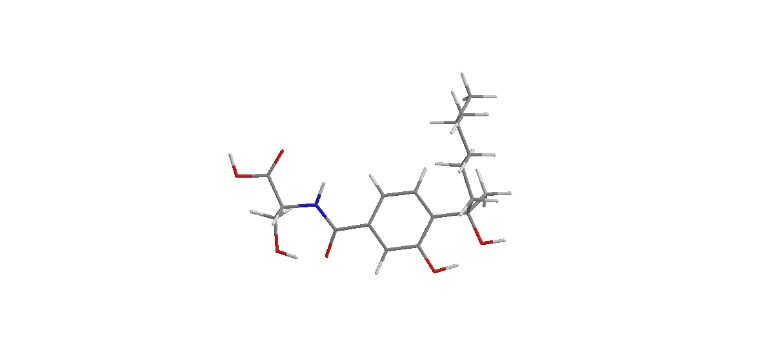


**N7**c-1 **N7**c-2 **N7**c-3

Optimized conformers (≥ 1%) of **N7c** at the B3LYP/6-31+G (d) level with PCM model in MeOH.

Table S5. Important Thermodynamic Parameters and Conformational Analysis of **N7c**

| Conformations | E+ZPE | G | % |
| --- | --- | --- | --- |
| N7c-1 | -1716.85067 | -1207.87601 | 0.10 |
| N7c-2 | -1716.85072 | -1207.88284 | 99.20 |
| N7c-3 | -1716.85072 | -1207.87823 | 0.70 |

E+ZPE: total energy with zero-point energy; G: Gibbs free energy; P: conformational distributions calculated from relative Gibbs free energy.

Table S6. Optimized Cartesian Coordinate of compound **N7**c at B3LYP/6-31G (d, p) level in MeOH Using the PCM Model

| No. | Atom | **N7**c-2 | | |
| --- | --- | --- | --- | --- |
|  |  | X | Y | Z |
| 1 | C | 0.58922 | -0.90404 | 1.23894 |
| 2 | C | 1.19176 | -1.2327 | 0.01733 |
| 3 | C | -0.77201 | -1.14176 | 1.41837 |
| 4 | C | 0.42251 | -1.81896 | -0.99117 |
| 5 | C | -1.57247 | -1.69787 | 0.41293 |
| 6 | C | -2.89102 | 3.7335 | -0.08351 |
| 7 | C | -0.94274 | -2.05082 | -0.80757 |
| 8 | C | 2.63902 | -1.00517 | -0.25568 |
| 9 | C | -4.32168 | 3.17356 | -0.06734 |
| 10 | C | -4.39202 | 1.71514 | -0.56244 |
| 11 | C | -3.08709 | -1.85412 | 0.58502 |
| 12 | C | 5.11126 | 0.91027 | -0.85285 |
| 13 | C | -3.6354 | 0.69782 | 0.30535 |
| 14 | C | -5.25837 | 4.06565 | -0.89608 |
| 15 | C | 5.17094 | 0.89985 | 1.67994 |
| 16 | C | 4.72261 | 0.14074 | 0.42832 |
| 17 | C | -3.84386 | -0.74099 | -0.18732 |
| 18 | C | -3.53712 | -1.94157 | 2.04695 |
| 19 | H | -2.468 | -2.94884 | -1.47739 |
| 20 | H | -6.29312 | 3.70441 | -0.85587 |
| 21 | H | -4.38024 | -3.17494 | -0.14406 |
| 22 | H | -5.24791 | 5.09953 | -0.53071 |
| 23 | H | 0.88128 | -2.0983 | -1.93306 |
| 24 | H | -4.9514 | 4.08226 | -1.95028 |
| 25 | H | -2.21712 | 3.16402 | 0.56562 |
| 26 | H | -4.61345 | -2.14469 | 2.07943 |
| 27 | H | 5.21119 | -0.84055 | 0.3934 |
| 28 | H | -2.87613 | 4.77539 | 0.25817 |
| 29 | H | -4.67903 | 3.18845 | 0.97422 |
| 30 | H | -3.36378 | -1.01251 | 2.5959 |
| 31 | H | -3.0161 | -2.75524 | 2.55984 |
| 32 | H | -4.91666 | -0.98203 | -0.14108 |
| 33 | H | -3.55209 | -0.80905 | -1.2428 |
| 34 | H | 6.25828 | 1.03918 | 1.64165 |
| 35 | H | 4.92855 | 0.30821 | 2.56681 |
| 36 | H | 4.78574 | 2.67167 | 1.0488 |
| 37 | H | -1.21711 | -0.88338 | 2.37248 |
| 38 | H | 1.16939 | -0.49883 | 2.06141 |
| 39 | H | -2.56387 | 0.92914 | 0.30738 |
| 40 | H | -3.97868 | 0.79208 | 1.34437 |
| 41 | H | 4.37012 | -0.57209 | -1.80777 |
| 42 | H | 2.75613 | 0.59733 | 1.04184 |
| 43 | H | -5.44901 | 1.41561 | -0.61381 |
| 44 | H | -4.01136 | 1.66854 | -1.5943 |
| 45 | H | -2.47571 | 3.71058 | -1.10004 |
| 46 | N | 3.27757 | -0.07284 | 0.48901 |
| 47 | O | 3.24791 | -1.64638 | -1.13978 |
| 48 | O | -3.41383 | -3.13421 | -0.04151 |
| 49 | O | 4.48305 | 2.13696 | 1.80597 |
| 50 | O | -1.61842 | -2.60854 | -1.84846 |
| 51 | O | 4.85849 | 0.28262 | -1.99923 |
| 52 | O | 5.61851 | 2.01863 | -0.82929 |

Fig. S14 Structure of **N7**d

**
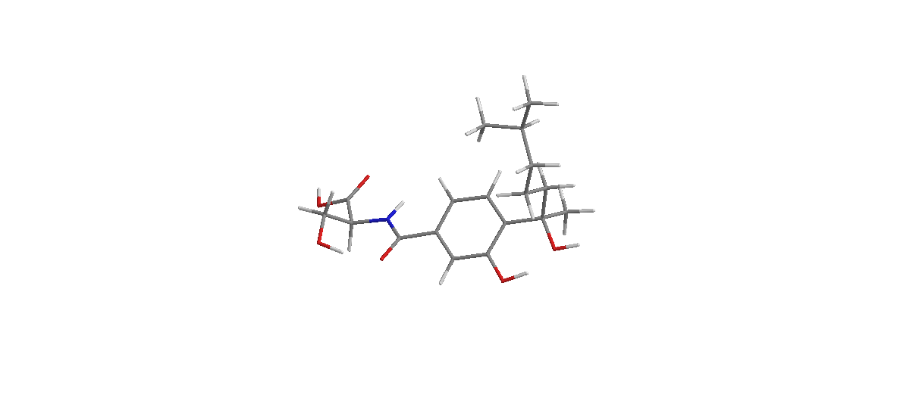

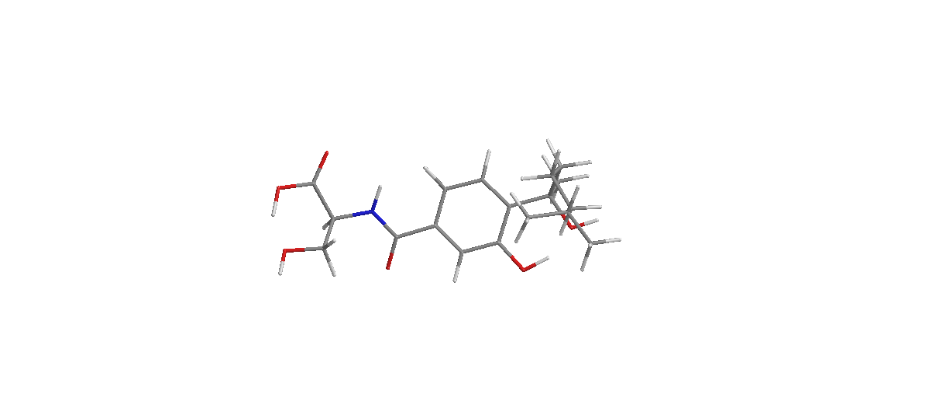

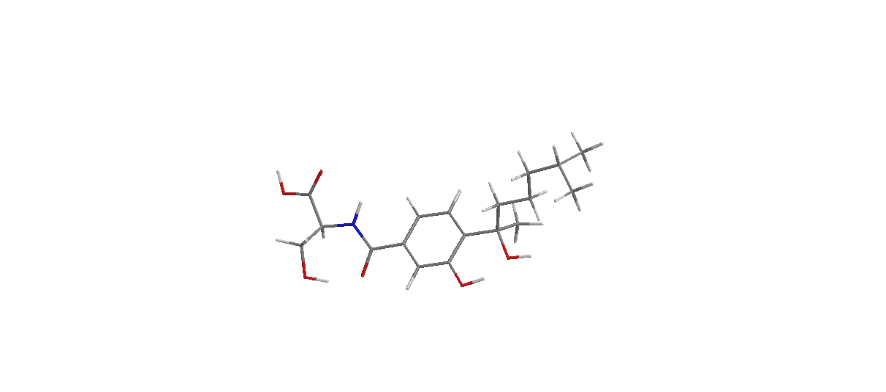
**

**N7**d-1 **N7**d-2 **N7d**-3

**
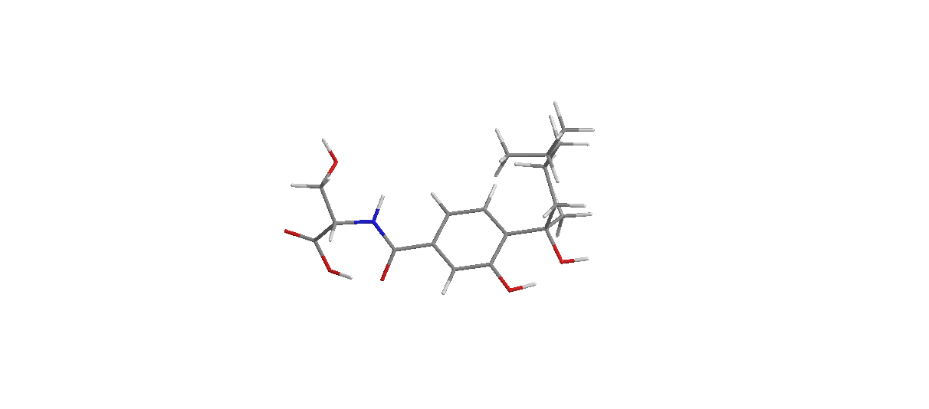
**

**N7d**-4

Optimized conformers (≥ 1%) of **N7d** at the B3LYP/6-31+G (d) level with PCM model in MeOH.

Table S7. Important Thermodynamic Parameters and Conformational Analysis of **N7d**

| Conformations | E+ZPE | G | % |
| --- | --- | --- | --- |
| N7d-1 | -1717.53727 | -1207.87694 | 41.10 |
| N7d-2 | -1717.53727 | -1207.87508 | 5.70 |
| N7d-3 | -1717.53727 | -1207.87625 | 19.80 |
| N7d-4 | -1716.53727 | -1207.87674 | 33.30 |

E+ZPE: total energy with zero-point energy; G: Gibbs free energy; P: conformational distributions calculated from relative Gibbs free energy.

Table S8. Optimized Cartesian Coordinate of compound **N7**d at B3LYP/6-31G (d, p) level in MeOH Using the PCM Model

| No. | Atom | **N7**d-1 | | | **N7**d-2 | | |
| --- | --- | --- | --- | --- | --- | --- | --- |
|  |  | X | Y | Z | X | Y | Z |
| 1 | C | 0.66968 | -0.54183 | 0.87158 | -0.73196 | -1.41752 | -0.36833 |
| 2 | C | 1.1475 | -0.47328 | -0.44492 | -1.0292 | -0.50716 | 0.65619 |
| 3 | C | -0.61634 | -1.0184 | 1.10871 | 0.57597 | -1.86271 | -0.5294 |
| 4 | C | 0.31219 | -0.86916 | -1.48885 | 0.00008 | -0.07277 | 1.48943 |
| 5 | C | -1.46985 | -1.44343 | 0.07958 | 1.6332 | -1.4339 | 0.28921 |
| 6 | C | -3.55446 | 3.10093 | 0.57808 | 3.52315 | 3.25717 | -0.85655 |
| 7 | C | -0.98088 | -1.34855 | -1.24761 | 1.32013 | -0.50712 | 1.31425 |
| 8 | C | 2.51504 | 0.02382 | -0.80287 | -2.40866 | 0.01551 | 0.93277 |
| 9 | C | -4.86605 | 2.31386 | 0.72095 | 4.81296 | 2.42583 | -0.93751 |
| 10 | C | -5.07864 | 1.30721 | -0.42896 | 4.58942 | 0.95314 | -0.53811 |
| 11 | C | -2.89293 | -1.90863 | 0.4267 | 3.04542 | -1.9628 | -0.0081 |
| 12 | C | 5.56725 | -0.0973 | 1.27591 | -5.49803 | -0.43864 | -1.02005 |
| 13 | C | -3.99825 | 0.22617 | -0.60997 | 3.62344 | 0.1874 | -1.45802 |
| 14 | C | -6.06386 | 3.27214 | 0.80906 | 5.91538 | 3.05539 | -0.0728 |
| 15 | C | 5.13831 | 1.77875 | -0.41154 | -4.9728 | 1.78115 | 0.12988 |
| 16 | C | 4.85956 | 0.301 | -0.01512 | -4.7 | 0.26496 | 0.08831 |
| 17 | C | -3.85056 | -0.7031 | 0.60279 | 3.61709 | -1.34483 | -1.30993 |
| 18 | C | -2.92752 | -2.79266 | 1.68461 | 3.06156 | -3.49956 | -0.11103 |
| 19 | H | -2.52518 | -2.1292 | -2.01837 | 3.08955 | -0.43734 | 1.99249 |
| 20 | H | -5.95415 | 3.9695 | 1.64825 | 6.11397 | 4.09069 | -0.37495 |
| 21 | H | -4.30712 | -2.84277 | -0.61197 | 4.81562 | -1.62974 | 0.83698 |
| 22 | H | -7.00412 | 2.72448 | 0.94697 | 6.85482 | 2.49501 | -0.15266 |
| 23 | H | 0.66063 | -0.80924 | -2.51386 | -0.21466 | 0.62319 | 2.29289 |
| 24 | H | -6.15635 | 3.86745 | -0.10905 | 5.62333 | 3.0683 | 0.98566 |
| 25 | H | -3.44655 | 3.82977 | 1.3903 | 3.11602 | 3.24506 | 0.16342 |
| 26 | H | -2.67518 | -2.23727 | 2.59198 | 2.45828 | -3.87001 | -0.94426 |
| 27 | H | 5.28036 | -0.3192 | -0.81915 | -5.03287 | -0.14171 | 1.05375 |
| 28 | H | -3.53273 | 3.65391 | -0.37056 | 2.7467 | 2.87905 | -1.53041 |
| 29 | H | -4.82297 | 1.75462 | 1.66722 | 5.15799 | 2.43769 | -1.98334 |
| 30 | H | -3.93942 | -3.1921 | 1.81564 | 4.09101 | -3.84204 | -0.26497 |
| 31 | H | -2.23382 | -3.632 | 1.58056 | 2.68486 | -3.94034 | 0.81667 |
| 32 | H | -3.50387 | -0.13405 | 1.47352 | 3.03878 | -1.7673 | -2.14067 |
| 33 | H | -4.83868 | -1.1046 | 0.87707 | 4.641 | -1.73049 | -1.4345 |
| 34 | H | 6.16907 | 2.0285 | -0.14744 | -4.77535 | 2.23605 | -0.84776 |
| 35 | H | 4.46686 | 2.42119 | 0.17962 | -4.31608 | 2.2324 | 0.87839 |
| 36 | H | 4.16565 | 1.55682 | -2.05987 | -6.63999 | 2.83653 | 0.28132 |
| 37 | H | -0.96747 | -1.04915 | 2.13464 | 0.78014 | -2.57168 | -1.32536 |
| 38 | H | 1.26497 | -0.19787 | 1.71142 | -1.50388 | -1.81704 | -1.01852 |
| 39 | H | -4.25887 | -0.37095 | -1.49253 | 2.60165 | 0.56486 | -1.33316 |
| 40 | H | -3.03273 | 0.6946 | -0.83443 | 3.89879 | 0.40114 | -2.5005 |
| 41 | H | 7.28738 | -0.19974 | 2.03968 | -6.96237 | 0.65308 | -0.48044 |
| 42 | H | 3.23424 | -0.45407 | 1.06281 | -3.06332 | -0.49188 | -0.97036 |
| 43 | H | -5.16348 | 1.87078 | -1.36992 | 4.22899 | 0.92243 | 0.49738 |
| 44 | H | -6.05003 | 0.81232 | -0.2829 | 5.56503 | 0.4436 | -0.54422 |
| 45 | H | -2.67543 | 2.44737 | 0.60311 | 3.71468 | 4.30276 | -1.12617 |
| 46 | N | 3.44826 | -0.01048 | 0.17681 | -3.29779 | -0.03264 | -0.09963 |
| 47 | O | 2.76468 | 0.43935 | -1.94881 | -2.72151 | 0.48711 | 2.03097 |
| 48 | O | -3.34284 | -2.74465 | -0.67768 | 3.89012 | -1.62853 | 1.1282 |
| 49 | O | 5.00753 | 1.9931 | -1.80024 | -6.35166 | 1.93802 | 0.50866 |
| 50 | O | -1.70572 | -1.68866 | -2.34595 | 2.23514 | 0.01991 | 2.17281 |
| 51 | O | 6.89277 | 0.07699 | 1.18965 | -6.78789 | -0.10452 | -1.1014 |
| 52 | O | 5.00358 | -0.53249 | 2.26062 | -5.00058 | -1.25263 | -1.77307 |

| No. | Atom | **N7**d-3 | | | **N7**d-4 | | |
| --- | --- | --- | --- | --- | --- | --- | --- |
|  |  | X | Y | Z | X | Y | Z |
| 1 | C | 1.01603 | 0.38497 | 0.56258 | 0.64758 | -0.71392 | 0.92736 |
| 2 | C | 1.77357 | -0.73938 | 0.20881 | 1.08023 | -0.19389 | -0.29971 |
| 3 | C | -0.37553 | 0.30648 | 0.56121 | -0.60286 | -1.32358 | 1.01249 |
| 4 | C | 1.11636 | -1.92325 | -0.1327 | 0.24986 | -0.29682 | -1.41872 |
| 5 | C | -1.05862 | -0.86552 | 0.21426 | -1.45604 | -1.43597 | -0.09222 |
| 6 | C | -7.38557 | 1.21197 | -1.65148 | -5.97082 | 3.2028 | 0.06153 |
| 7 | C | -0.27899 | -1.99634 | -0.13735 | -0.99931 | -0.9155 | -1.32978 |
| 8 | C | 3.27129 | -0.74532 | 0.18824 | 2.39923 | 0.47935 | -0.47559 |
| 9 | C | -6.94055 | 1.8436 | -0.32276 | -4.80102 | 2.20687 | 0.02937 |
| 10 | C | -5.41098 | 1.77047 | -0.11293 | -4.94541 | 1.18759 | 1.17864 |
| 11 | C | -2.58822 | -0.96379 | 0.31093 | -2.86772 | -2.01933 | 0.03414 |
| 12 | C | 5.49312 | 2.20832 | 0.08738 | 5.62706 | 0.50411 | -0.63119 |
| 13 | C | -4.81077 | 0.35951 | 0.00097 | -3.89243 | 0.06521 | 1.24676 |
| 14 | C | -7.72818 | 1.25853 | 0.86029 | -3.45747 | 2.9508 | 0.0685 |
| 15 | C | 6.17156 | -0.02607 | -0.93955 | 5.22874 | 0.69386 | 1.88925 |
| 16 | C | 5.30819 | 0.69563 | 0.1354 | 4.64514 | 0.90946 | 0.49533 |
| 17 | C | -3.28419 | 0.40955 | 0.17427 | -3.92681 | -0.88384 | 0.04039 |
| 18 | C | -2.96071 | -1.67299 | 1.62731 | -3.03928 | -2.97478 | 1.21913 |
| 19 | H | -1.7783 | -3.01101 | -0.68531 | -2.43425 | -1.66087 | -2.31305 |
| 20 | H | -7.41554 | 1.71049 | 1.80969 | -2.60549 | 2.2724 | -0.05052 |
| 21 | H | -3.90485 | -2.10239 | -0.64816 | -4.00672 | -3.03224 | -1.24039 |
| 22 | H | -7.5883 | 0.17389 | 0.94383 | -3.33302 | 3.48084 | 1.0224 |
| 23 | H | 1.68838 | -2.80295 | -0.40601 | 0.57505 | 0.09496 | -2.3759 |
| 24 | H | -8.80278 | 1.44084 | 0.74013 | -3.40072 | 3.69461 | -0.73519 |
| 25 | H | -7.21873 | 0.12792 | -1.66106 | -5.96329 | 3.7858 | 0.99216 |
| 26 | H | -2.65338 | -1.06789 | 2.4858 | -2.94337 | -2.47124 | 2.18428 |
| 27 | H | 5.67209 | 0.3484 | 1.11139 | 4.44484 | 1.98003 | 0.35746 |
| 28 | H | -6.83901 | 1.64191 | -2.49986 | -6.93661 | 2.68659 | -0.00024 |
| 29 | H | -7.18504 | 2.915 | -0.37598 | -4.85898 | 1.66499 | -0.9259 |
| 30 | H | -4.04225 | -1.8365 | 1.69762 | -4.0405 | -3.41846 | 1.17939 |
| 31 | H | -2.46129 | -2.64417 | 1.69223 | -2.30151 | -3.78081 | 1.16922 |
| 32 | H | -2.84783 | 0.92969 | -0.68768 | -4.9198 | -1.35609 | -0.01225 |
| 33 | H | -3.05202 | 1.01197 | 1.0608 | -3.80003 | -0.31521 | -0.88745 |
| 34 | H | 7.13447 | 0.48521 | -1.02171 | 4.63071 | 1.26104 | 2.61364 |
| 35 | H | 5.65258 | 0.06163 | -1.9071 | 6.25775 | 1.06985 | 1.90635 |
| 36 | H | 5.58483 | -1.75074 | -0.31075 | 5.33766 | -0.82946 | 3.11266 |
| 37 | H | -0.93718 | 1.18569 | 0.85524 | -0.92302 | -1.70668 | 1.97484 |
| 38 | H | 1.49052 | 1.30874 | 0.87798 | 1.25144 | -0.618 | 1.82372 |
| 39 | H | -5.27046 | -0.16641 | 0.84661 | -2.89059 | 0.49924 | 1.34686 |
| 40 | H | -5.05432 | -0.21371 | -0.9019 | -4.07048 | -0.50629 | 2.16603 |
| 41 | H | 6.81869 | 3.5344 | 0.28037 | 4.19916 | 0.95993 | -1.84045 |
| 42 | H | 3.31966 | 1.28083 | -0.16246 | 3.2755 | -0.57552 | 1.06863 |
| 43 | H | -5.15694 | 2.33521 | 0.79587 | -5.94394 | 0.73062 | 1.11735 |
| 44 | H | -4.92012 | 2.29497 | -0.94554 | -4.92161 | 1.73773 | 2.13091 |
| 45 | H | -8.45566 | 1.37971 | -1.82254 | -5.91304 | 3.91016 | -0.77439 |
| 46 | N | 3.87578 | 0.45327 | 0.02008 | 3.3705 | 0.19673 | 0.41901 |
| 47 | O | 3.90817 | -1.80798 | 0.30809 | 2.60675 | 1.2703 | -1.42244 |
| 48 | O | -2.99672 | -1.8002 | -0.81565 | -3.06226 | -2.80793 | -1.18181 |
| 49 | O | 6.44537 | -1.36537 | -0.59229 | 5.17379 | -0.70423 | 2.16526 |
| 50 | O | -0.83073 | -3.19316 | -0.47684 | -1.73838 | -0.97807 | -2.47045 |
| 51 | O | 6.76214 | 2.5603 | 0.33423 | 5.1397 | 0.61574 | -1.87087 |
| 52 | O | 4.60245 | 3.00119 | -0.14646 | 6.77155 | 0.15549 | -0.42936 |


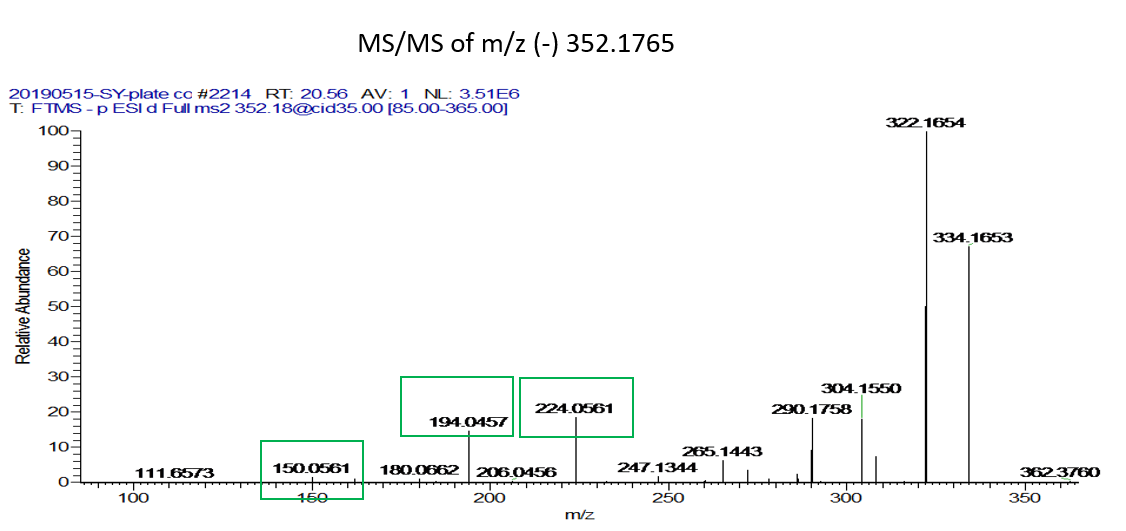


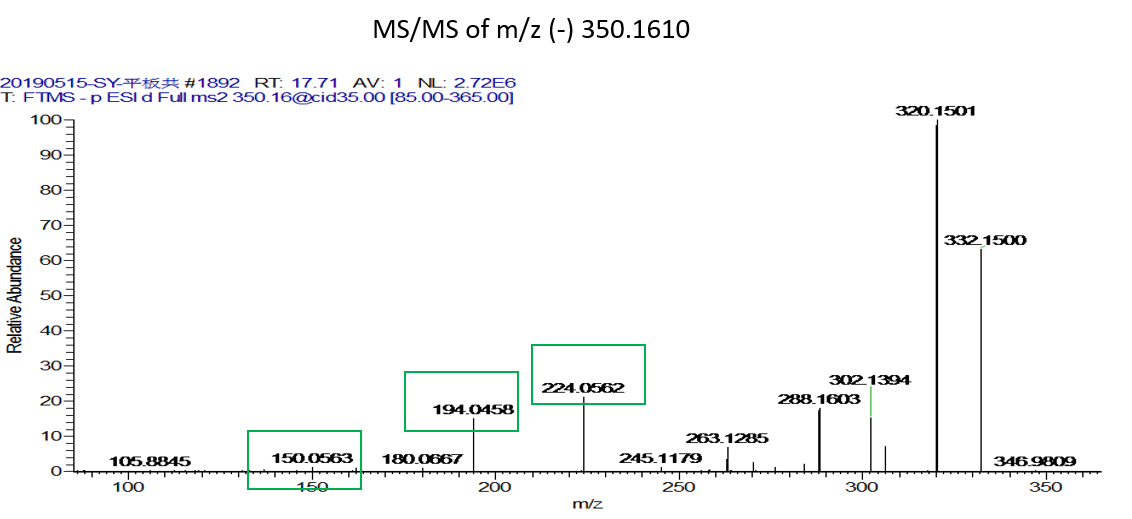

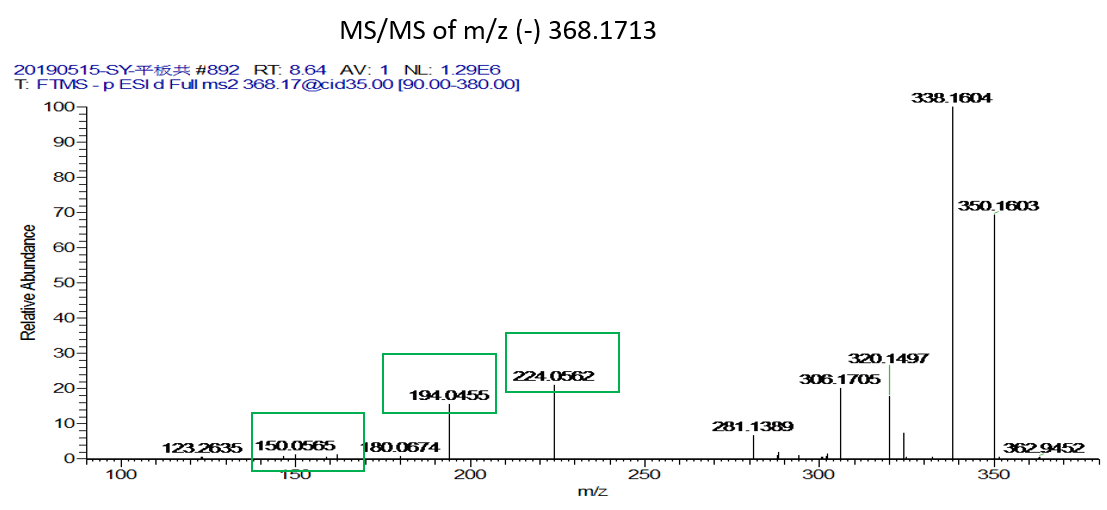


Fig. S15 MS/MS spectrum of induced features involved in molecular network analysis (**N7**: m/z 353.1765; **N6**: m/z 351.1610; **N9**: m/z 369.1713)

Fig. S16 Positive ESI-HRMS spectrum of **N20**


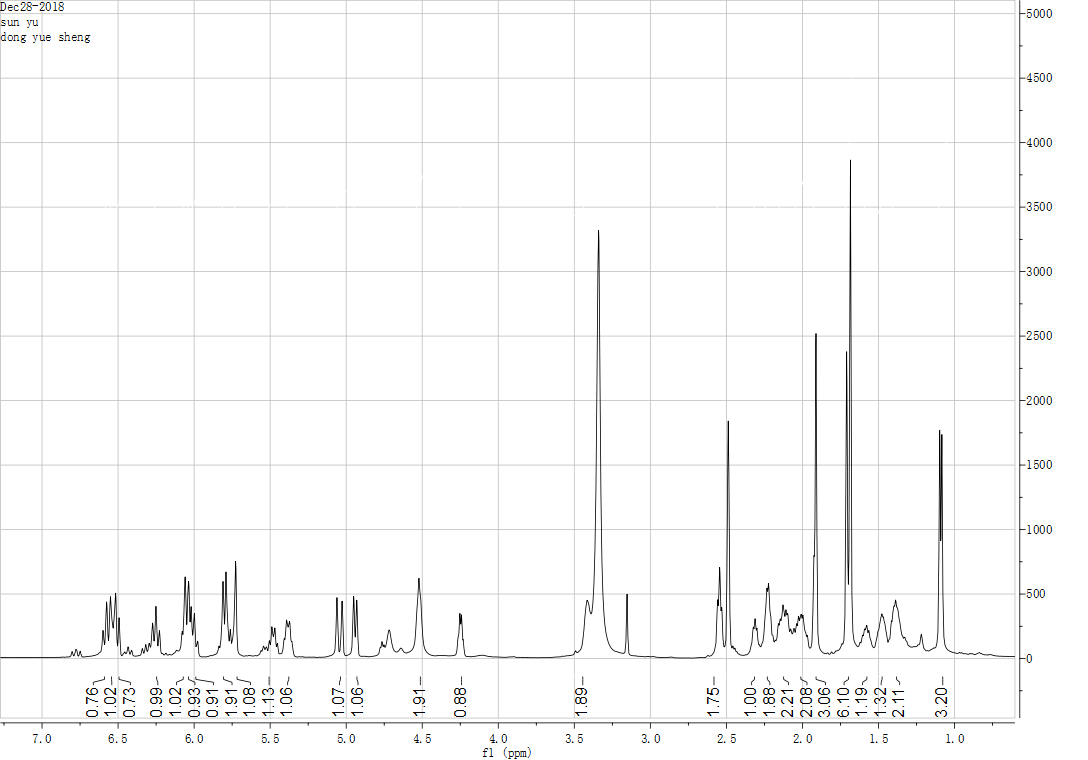


Fig. S17 ^1^H-spectrum of compound **N20** (DMSO-*d6*)


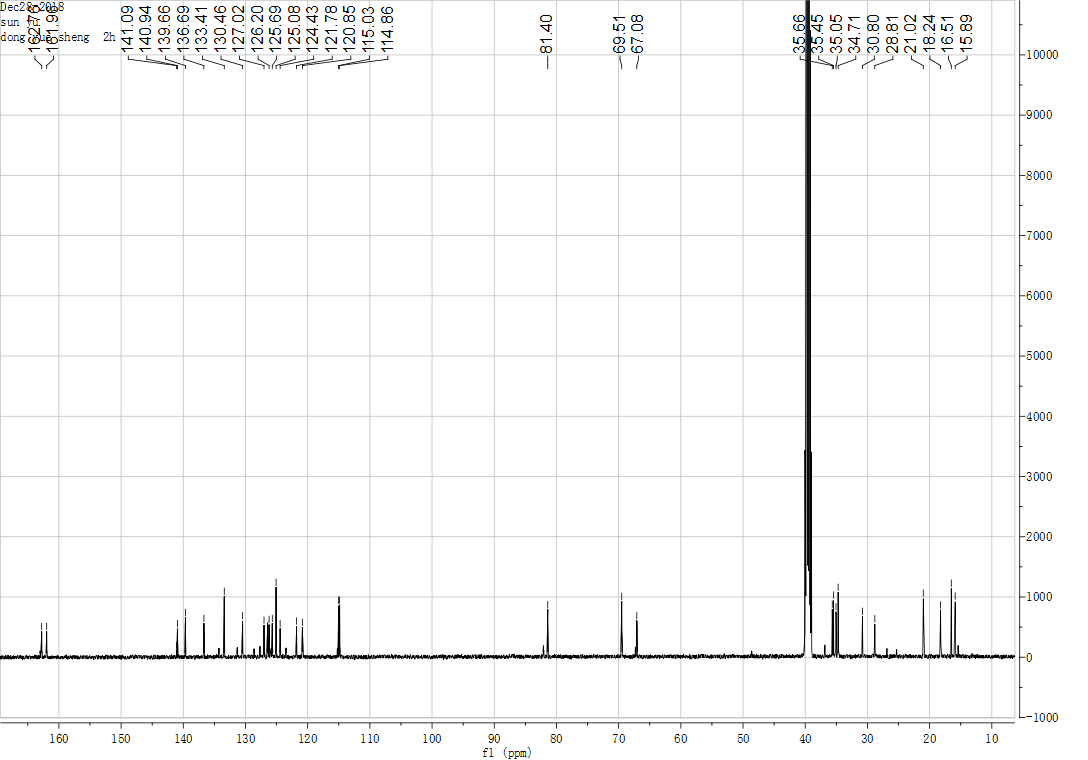


Fig. S18 ^13^C-spectrum of compound **N20** (DMSO-*d6*)


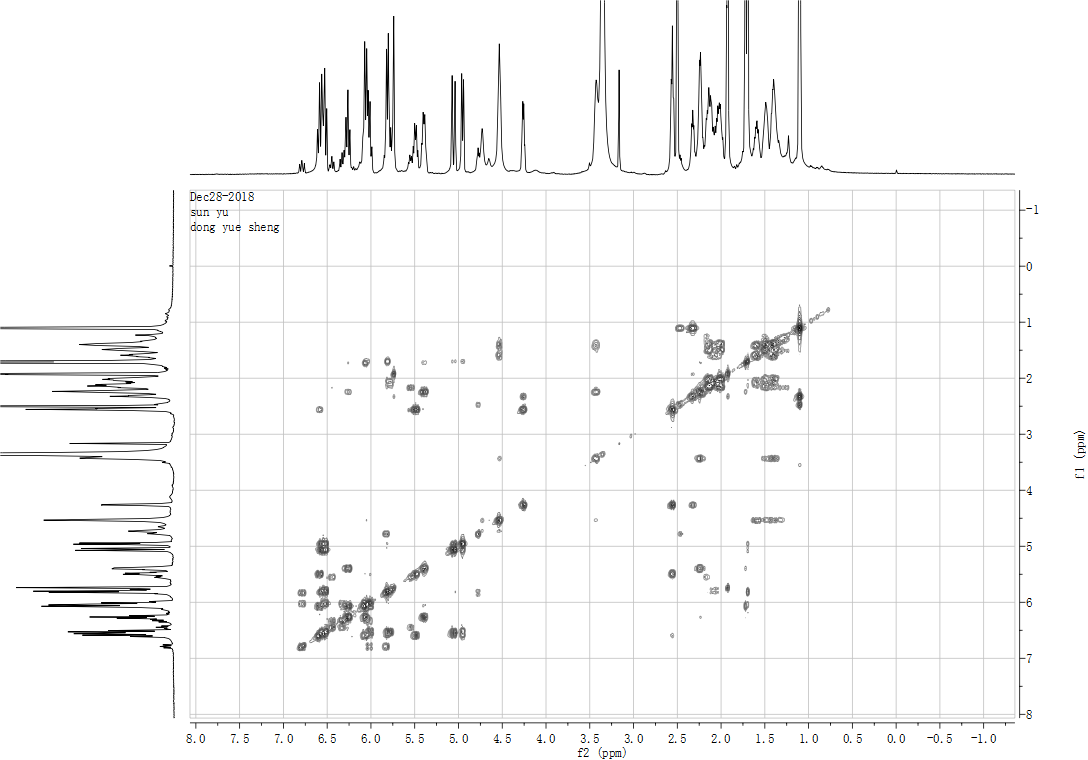


Fig. S19 COSY spectrum of compound **N20** (DMSO-*d6*)


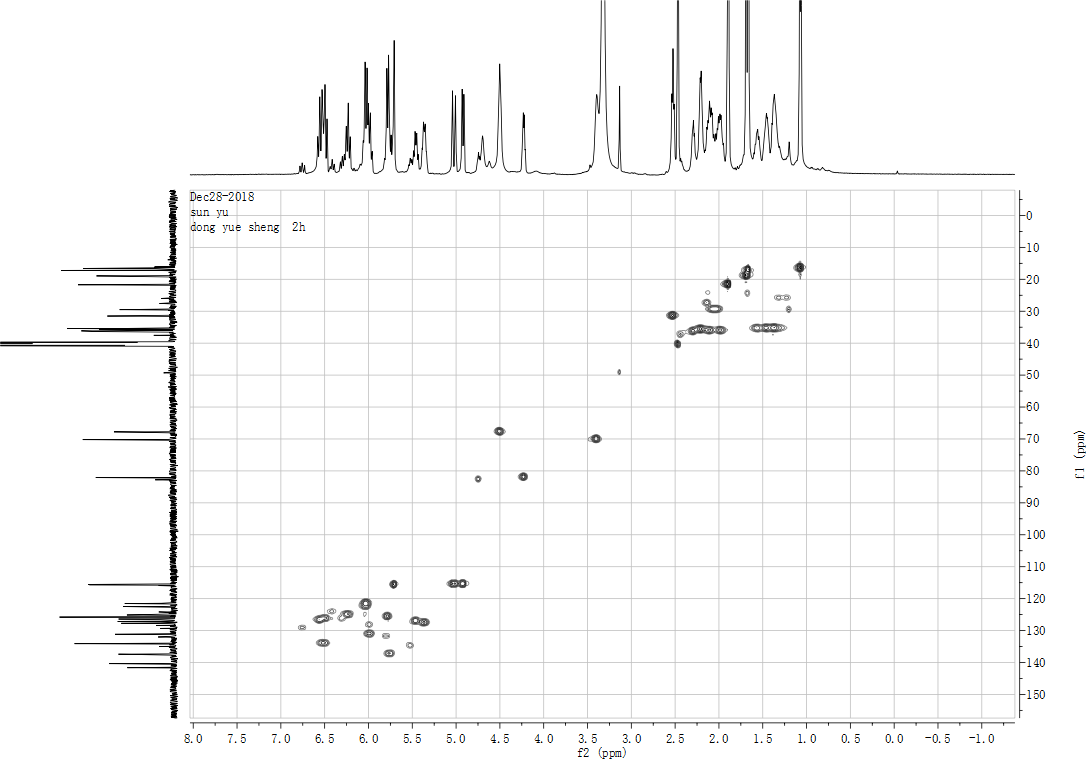


Fig. S20 HMQC spectrum of compound **N20** (DMSO-*d6*)


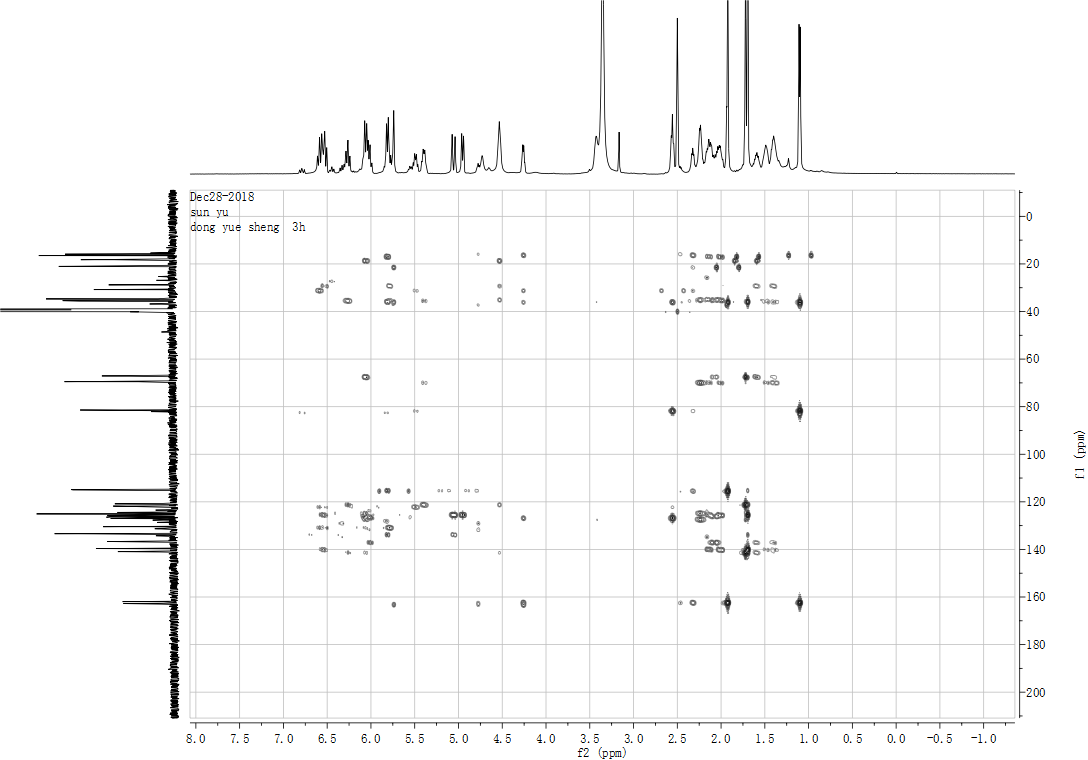


Fig. S21 HMBC spectrum of compound **N20** (DMSO-*d6*)


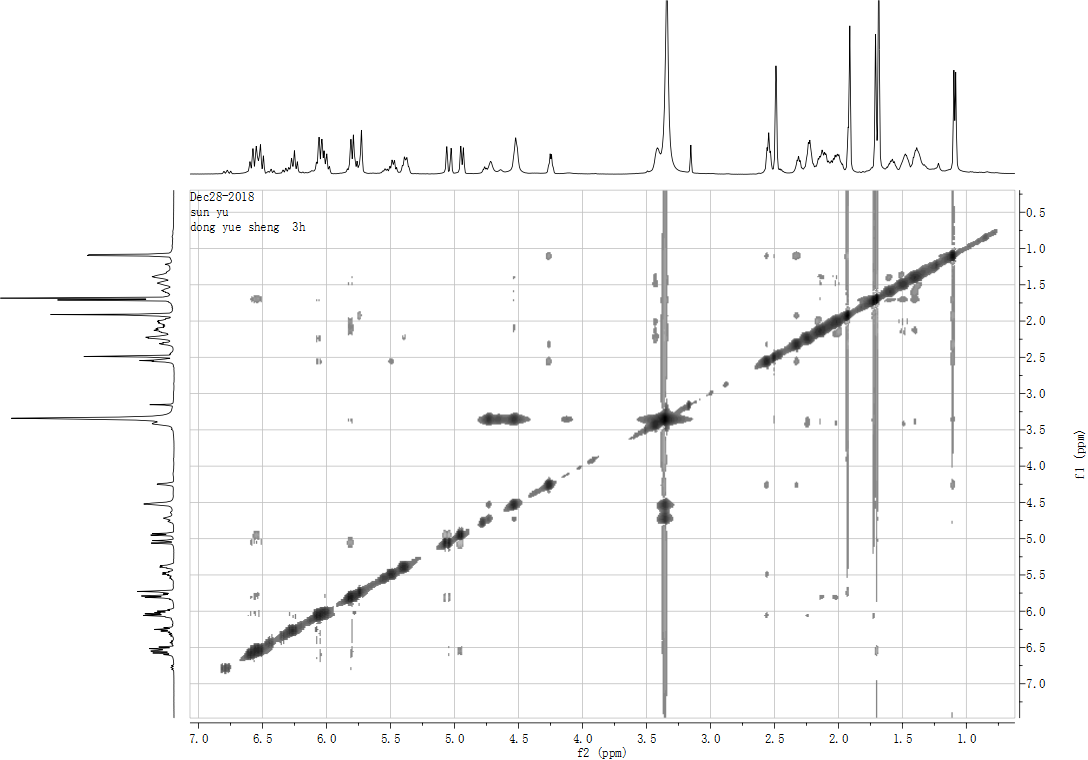


Fig. S22 NOE spectrum of compound **N20** (DMSO-*d6*)

Fig. S23 Partial enlarged view of the NOE spectrum of compound **N20** (DMSO-*d6*)

Fig. 24 Structures of 25 compounds

**Reference**

1. Fang MJ, Fang H, Li WJ, Huang DM, Wu Z, Zhao YF. A new diphenyl ether from *Phoma sp.* strain, SHZK-2. Nat Prod Res. 2012;26:1224-1228.

2. Shinji K, Takanori M, Junsuke M, Kazuaki T, Noboru T. Isolation and absolute stereochemistry of optically active sydonic acid from *Glonium sp.* (Hysteriales, Ascomycota). Biosci Biotech Bioch. 2009;73:203-204.

3. Li XD, Li XM, Xu GM, Zhang P, Wang BG. Antimicrobial Phenolic Bisabolanes and Related Derivatives from *Penicillium aculeatum* SD-321, a Deep Sea Sediment-Derived Fungus. J Nat Prod. 2015;78:844-849.

4. Wang CY, Liu YF, Cao F, Wang CY. Bisabolane-Type Sesquiterpenoids from a Gorgonian-Derived *Aspergillus sp.* Fungus Induced by DNA Methyltransferase Inhibitor. Chem Nat Compd+. 2016;52:1129-1132.

5. He S, Wang H, Yan X, Zhu P, Chen J, Yang R. Preparative isolation and purification of macrolactin antibiotics from marine bacterium *Bacillus amyloliquefaciens* using high-speed counter-current chromatography in stepwise elution mode. J Chromatogr A. 2013;1272:15-19.
